# The global inverse fractional conductivity problem

**DOI:** 10.1007/s00526-026-03401-6

**Published:** 2026-09-08

**Authors:** Giovanni Covi, Jesse Railo, Philipp Zimmermann

**Affiliations:** 1 Institut fur Angewandte Mathematik, Ruprecht-Karls-Universität Heidelberg, Im Neuenheimer Feld 205, 69120 Heidelberg, Germany; 2 University of Jyväskylä, Department of Mathematics and Statistics, Seminaarinkatu 15, FI-40014 University of Jyväskylä, PO Box 35 Finland; 3 Department of Pure Mathematics and Mathematical Statistics, University of Cambridge, Cambridge, CB3 0WB UK; 4 Computational Engineering, School of Engineering Sciences, Lappeenranta-Lahti University of Technology LUT, Lappeenranta, Finland; 5 Department of Mathematics, ETH Zurich, Zürich, Switzerland; 6 Institute of Mathematics, École Polytechnique Fédérale de Lausanne (EPFL), Lausanne, Switzerland

**Keywords:** 35R30, 26A33, 42B37

## Abstract

We prove *global* uniqueness for an inverse problem for the fractional conductivity equation on domains that are bounded in one direction. The conductivities are assumed to be nontrivial in the exterior of the domain and isotropic, while the data is given in the form of partial Dirichlet-to-Neumann (DN) maps measured in nondisjoint open subsets of the exterior. This can be seen as the fractional counterpart of the classical inverse conductivity problem. The proof is based on a unique continuation property (UCP) for the DN maps and a novel exterior determination method from the partial exterior DN maps, which is a nonlocal analog of the classical boundary determination method by Kohn and Vogelius. Exterior determination is achieved independently of the UCP and despite the nonlocality of the equation.

## Introduction

In this article we study the uniqueness, reconstruction and stability properties of the inverse problem for the fractional conductivity equation

1 $$\begin{aligned} \begin{aligned} \text{ div}_s(\Theta _{\gamma }\nabla ^s u)&= 0\quad \text {in}\quad \Omega ,\\ u&= f\quad \text {in}\quad \Omega _e = {\mathbb {R}}^n \setminus \overline{\Omega }, \end{aligned} \end{aligned}$$

for a possibly unbounded open set $\Omega \subset {\mathbb {R}}^n$ which is bounded in one direction, and 0<s<min(1,n/2). We say that an open set Ω is bounded in one direction if it is contained between two parallel hyperplanes, whose normal determines the direction of boundedness. The rigorous definition of boundedness in one direction is given in Definition 3.1. Here $\gamma \in L^\infty ({\mathbb {R}}^n)$ is a positive conductivity, and $\Theta _{\gamma }(x,y):=\gamma ^{1/2}(x)\gamma ^{1/2}(y)\textbf{1}_{n\times n}$ for $x,y\in {\mathbb {R}}^n$ denotes the conductivity matrix. The fractional gradient of order *s* is the bounded linear operator $\nabla ^s:H^s({\mathbb {R}}^n)\rightarrow L^2({\mathbb {R}}^{2n};{\mathbb {R}}^n)$ given by

$$ \nabla ^su(x,y)=\sqrt{\frac{C_{n,s}}{2}}\frac{u(x)-u(y)}{|x-y|^{n/2+s+1}}(x-y), $$

where $C_{n,s}>0$ is a constant. This operator appears in the study of weighted long jump random walk models as in [16, Section 5] and [13], analogously to the model of nonlocal diffusion with constant coefficient [10, Chapter 2]. Furthermore, $\nabla ^s$ is naturally associated with the $L^2$ Gagliardo seminorm, cf. the identity (2) with a≡1. We denote the adjoint of $\nabla ^s$ by $\hbox {div}_s$. Such operators were first introduced in the fractional calculus theory developed in [20], and later studied in [16, 49]. They enjoy good mapping properties and verify the equality $\hbox {div}_s\nabla ^s = (-\Delta )^s$. We use the notation

$$\begin{aligned} m_{\gamma }:=\gamma ^{1/2}-1 \end{aligned}$$

for the background deviation and $\Lambda _\gamma$ for the exterior DN map associated to (1).

The fractional conductivity operator $\text{ div}_s(\Theta _{\gamma }\nabla ^s\cdot )$ in (1) is an isotropic special case of more general nonlocal operators given weakly by bilinear forms of the type:

$$\begin{aligned} B_K(f,g) =\int _{{\mathbb {R}}^{2n}} K(x,y)\nabla ^s f \cdot \nabla ^s g\,dxdy, \quad K\in L^{\infty }({\mathbb {R}}^{2n};{\mathbb {R}}^{n\times n}). \end{aligned}$$

In the isotropic case $K(x,y) =a(x,y)\textbf{1}_{n\times n}$ for some $a\in L^{\infty }({\mathbb {R}}^{2n})$, this reduces into the form

2 $$\begin{aligned} B_a(f,g)=\frac{C_{n,s}}{2}\int _{{\mathbb {R}}^{2n}} \frac{a(x,y)}{\left\lvert x-y \right\rvert ^{n+2s}} (f(x)-f(y))(g(x)-g(y))\,dxdy \end{aligned}$$

where *a*(*x*, *y*) is the symbol of the related operator. In this article, we characterize the uniqueness of a related partial data inverse problem with the symbols of product type, i.e. $a(x,y)=\sigma (x)\sigma (y)$ for a real function $\sigma \in L^\infty ({\mathbb {R}}^n)$ with $\sigma \ge \sigma _0 > 0$ and sufficient regularity. We denote $\sigma =\gamma ^{1/2}$ in this article as then the related fractional conductivity operator converges to the classical conductivity operator in the sense of distributions as s↑1 (cf. [16]). Ghosh and Uhlmann introduced a novel approximation result in [27], which makes it possible to reduce the exterior Cauchy data of fractional order operators, generated by the heat semigroups, to the corresponding boundary Cauchy data of the associated elliptic operators. See also the survey [42] about different properties of nonlocal elliptic equations on bounded domains.

Our work almost completely characterizes global uniqueness for the related partial data inverse problems. We rely on many ideas from the recent works [16, 26, 45, 48, 49] and the classical work [34] of Kohn and Vogelius. On the background, somewhat transparently in our article, one utilizes Carleman estimates for the Caffarelli–Silvestre extensions [19, 47] via the unique continuation property (UCP) of the fractional Laplacians (see the seminal work [26] by Ghosh, Salo and Uhlmann for details).

Both the UCP and a comprehensive analysis of many different aspects of the fractional conductivity equation in [16, 48, 49] are essential in our proof. In the present work, we have achieved a *global* uniqueness result for an inverse problem of the nonlocal variable coefficient operators (2) with *nontrivial coefficients in the whole Euclidean space*. This result is formulated in Theorem 1.3. A construction of sequences of special solutions whose Dirichlet energies can be concentrated in the limit, despite the nonlocal nature of the studied operators, is an indispensable part of our theory. Though possible generalizations, sharper regularity assumptions, global stability, reconstruction, and many other natural questions remain viable research directions, the analysis of the model problem (1) may drive research towards new objectives beyond a standard application of the UCP and enriches the already vast mathematical theory of nonlocal inverse problems.

The theory of nonlocal inverse problems has been widely developed in the last years [50], but it has focused on the determination of additive perturbations in Ω or operators with trivial a priori known coefficients in the exterior $\Omega _e$ (see e.g. [15, 17, 27, 37, 49] and the references therein). The recent work [24], solving a geometric inverse problem for the fractional spectral Laplacian on closed Riemannian manifolds, is a notable exception where the nonlocal operator is assumed to be nontrivial in the whole space. The data in [24] is given by local source-to-solution maps of globally defined fractional (spectral) Poisson’s equations, and the measurements take place in a fixed support of controllable sources. Analogously, in our setting the measurements take place in a fixed support of controllable exterior values. However, these two inverse problems are quite different, as ${\mathbb {R}}^n$ is an open manifold, the fractional spectral Laplacians depend on the set Ω (whereas the Fourier multiplier $(-\Delta )^s$ or nonlocal operators with the weak form (2) do not), and exterior value and interior source problems model different geometric measurement settings.

We describe our main results next. We have obtained the following results dealing with the invariance of data (for conductivities which a priori agree on the sets where measurements are performed) and exterior determination:

### Theorem 1.1

(UCP of the exterior DN map and invariance of data) Let $\Omega \subset {\mathbb {R}}^n$ be an open set which is bounded in one direction and 0<s<min(1,n/2). Assume that $\gamma _1,\gamma _2\in L^{\infty }({\mathbb {R}}^n)$ with background deviations $m_1,m_2$ satisfy $\gamma _1(x),\gamma _2(x)\ge \gamma _0>0$ and $m_1,m_2\in H^{2s,\frac{n}{2s}}({\mathbb {R}}^n)$. Moreover, assume that $m_0:=m_1-m_2\in H^s({\mathbb {R}}^n)$. Finally, assume that $W_1,W_2 \subset \Omega _e$ are nonempty open sets and that $\gamma _1|_{W_1\cup W_2} = \gamma _2|_{W_1\cup W_2}$ holds. Then

(i) if $W_1\cap W_2\ne \emptyset$, we have $\left. \Lambda _{\gamma _1}f\right| _{W_2}=\left. \Lambda _{\gamma _2}f\right| _{W_2}$ for all $f\in C_c^{\infty }(W_1)$ if and only if $\gamma _1=\gamma _2$ in ${\mathbb {R}}^n$.
(ii) if $W_1\cap W_2=\emptyset$, we have $\left. \Lambda _{\gamma _1}f\right| _{W_2}=\left. \Lambda _{\gamma _2}f\right| _{W_2}$ for all $f\in C_c^{\infty }(W_1)$ if and only if $m_1-m_2$ is the unique solution of
  $$\begin{aligned} \begin{aligned} (-\Delta )^sm-\frac{(-\Delta )^sm_1}{\gamma _1^{1/2}}m&=0\quad \text {in}\quad \Omega ,\\ m&=m_0\quad \text {in}\quad \Omega _e. \end{aligned} \end{aligned}$$

### Theorem 1.2

(Exterior determination) Let $\Omega \subset {\mathbb {R}}^n$ be an open set which is bounded in one direction and 0<s<min(1,n/2). Assume that $\gamma _1,\gamma _2\in L^{\infty }({\mathbb {R}}^n)$ with background deviations $m_1,m_2$ satisfy $\gamma _1(x),\gamma _2(x)\ge \gamma _0>0$ and $m_1,m_2\in H^{2s,\frac{n}{2s}}({\mathbb {R}}^n)$.

Suppose that $W \subset \Omega _e$ is a nonempty open set such that $\gamma _1,\gamma _2\in C(W)$. If $\left. \Lambda _{\gamma _1}f\right| _{W}=\left. \Lambda _{\gamma _2}f\right| _{W}$ for all $f\in C_c^{\infty }(W)$, then $\gamma _1=\gamma _2$ in *W*.

The proofs of Theorems 1.1 and 1.2 are given in Sections 4 and 5.3, respectively. Observe that none of Theorems 1.1 (i) and 1.2 in general implies the other. However, it is evident that Theorem 1.1 (i) with $W_1 = W_2:= W$ and Theorem 1.2 directly imply the following stronger global uniqueness theorem, which is the main result of our work:

### Theorem 1.3

(Global uniqueness) Let $\Omega \subset {\mathbb {R}}^n$ be an open set which is bounded in one direction and 0<s<min(1,n/2). Assume that $\gamma _1,\gamma _2\in L^{\infty }({\mathbb {R}}^n)$ with background deviations $m_1,m_2$ satisfy $\gamma _1(x),\gamma _2(x)\ge \gamma _0>0$ and $m_1,m_2\in H^{2s,\frac{n}{2s}}({\mathbb {R}}^n) \cap H^{s}({\mathbb {R}}^n)$. Suppose that $W \subset \Omega _e$ is a nonempty open set such that $\gamma _1,\gamma _2\in C(W)$. Then $\left. \Lambda _{\gamma _1}f\right| _{W}=\left. \Lambda _{\gamma _2}f\right| _{W}$ for all $f\in C_c^{\infty }(W)$ if and only if $\gamma _1=\gamma _2$ in ${\mathbb {R}}^n$.

We emphasize that our results hold in any dimension n≥1, without restrictions on s∈(0,1) if n≥2. On the other hand, if n=1, we restrict to the case s∈(0,1/2). This restriction is related to the multiplier and embedding results for Bessel potential spaces, which are used in our proof of Lemma 4.2. This leaves an interesting open question when n=1, even in the case of bounded intervals (*a*, *b*). However, it seems that assuming regular enough conductivities, e.g. the case (i) of Remark 1.8, would suffice to solve the issue. We do not address this problem here, but refer to [49] for a comprehensive treatment.

The case $W:= W_1 \cap W_2 \ne \emptyset$ is essentially the most general one for which the global uniqueness may hold for general partial data problems with the knowledge of $\Lambda _{\gamma _1}f|_{W_2}=\Lambda _{\gamma _2}f|_{W_2}$ for all $f \in C_c^\infty (W_1)$. When Ω is a bounded domain, $W_1 \cap W_2 = \emptyset$ and $\overline{W_1\cup W_2} \subset \Omega _e$, it is always possible to find counterexamples to the global uniqueness (see [48, Theorem 1.2]). Similar counterexamples are also possible on domains that are bounded in one direction as proved in [48, Theorem 1.3] whenever ${{\,\textrm{dist}\,}}(W_1\cup W_2,\Omega )>0$ and 0<s<min(1,n/4). There are still interesting open questions related to the existence of concrete counterexamples when these additional assumptions on Ω, $W_1$ and $W_2$ do not hold, but we believe that this is merely due to some technical difficulties to verify the right regularity of possible counterexample candidates. Therefore, it is expected that the uniqueness is always lost in the case (ii) of Theorem 1.1.

Our main result is complemented by the following two, which are proved in Section 5.3, and deal with the questions of stability and reconstruction in the exterior:

### Proposition 1.4

(Stability estimate) Let $\Omega \subset {\mathbb {R}}^n$ be an open set which is bounded in one direction and 0<s<min(1,n/2). Assume that $\gamma _1,\gamma _2\in L^{\infty }({\mathbb {R}}^n)$ with background deviations $m_1,m_2$ satisfy $\gamma _1(x),\gamma _2(x)\ge \gamma _0>0$ and $m_{1},m_2\in H^{2s,\frac{n}{2s}}({\mathbb {R}}^n)$. If $W \subset \Omega _e$ is a nonempty open set such that $\gamma _i\in C(W)$, i=1,2, then

$$\begin{aligned} \Vert \gamma _1-\gamma _2\Vert _{L^{\infty }(W)}\le 2^s\Vert \Lambda _{\gamma _1}-\Lambda _{\gamma _2}\Vert _{X\rightarrow X^*}. \end{aligned}$$

### Proposition 1.5

(Exterior reconstruction formula) Let $\Omega \subset {\mathbb {R}}^n$ be an open set which is bounded in one direction and 0<s<min(1,n/2). Assume that $\gamma \in L^{\infty }({\mathbb {R}}^n)$ with background deviation $m_{\gamma }$ satisfy $\gamma (x)\ge \gamma _0>0$ and $m_{\gamma }\in H^{2s,\frac{n}{2s}}({\mathbb {R}}^n)$. If $W \subset \Omega _e$ is a nonempty, bounded, open set such that $\gamma \in C(W)$, $x_0\in W$, then there exist $(\phi _N)_{N \in {\mathbb {N}}}\subset C_c^{\infty }(W)$ and solutions $(u_N)_{N \in {\mathbb {N}}}\subset H^s({\mathbb {R}}^n)$ of the homogeneous fractional conductivity equation with exterior values $\phi _N$ such that

$$ \gamma (x_0)=\lim _{N\rightarrow \infty }E_{\gamma }(u_N). $$

We make some remarks about our results and assumptions next.

### Remark 1.6

The continuity assumption in Theorem 1.2 can be replaced by the assumption that there exists $\widetilde{\gamma }_1,\widetilde{\gamma }_2 \in L^\infty ({\mathbb {R}}^n)$ such that $\widetilde{\gamma }_1,\widetilde{\gamma }_2$ are continuous a.e. in *W* and $\widetilde{\gamma }_1=\gamma _1, \widetilde{\gamma }_2=\gamma _2$ a.e. in *W*. In this case, the conclusion $\gamma _1 = \gamma _2$ holds a.e. in *W*. This follows from the proof of Theorem 1.2 with minor modifications. This observation carries over to Theorem 1.3, and Propositions 1.4 and 1.5.

### Remark 1.7

If δ>0 and one has that $m_\gamma \in H^{2s+\delta ,n/2s}({\mathbb {R}}^n) \cap H^s({\mathbb {R}}^n)$, then the Sobolev embeddings into Hölder spaces give that γ is continuous and Theorem 1.3 is known to apply. Therefore, the continuity assumption in exterior determination is not very restrictive in the light of the regularity assumptions in Theorem 1.1.

### Remark 1.8

*(Examples)* Let 0<c<1 be a small fixed parameter. Then the following conductivities satisfy the assumptions of Theorem 1.3:

(i) γ=h+1 for some $h \in C_c^\infty ({\mathbb {R}}^n)$ such that h≥c-1.
(ii) $\gamma = (m+1)^2 = m^2 +2m +1$ for some $m \in H^{2s,n/2s}({\mathbb {R}}^n) \cap H^{s}({\mathbb {R}}^n) \cap L^\infty ({\mathbb {R}}^n)$ such that m≥c-1 and m∈C(W). We have that $m^2 +2m \in H^{2s,n/2s}({\mathbb {R}}^n)$ by [1].

We organize the article as follows. In Section 2, we briefly compare the problem to the classical inverse conductivity problem. We recall preliminaries on Bessel potential spaces, the fractional Laplacians, and the fractional Schrödinger and fractional conductivity equations in Section 3. We prove Theorem 1.1 in Section 4. We prove elliptic energy estimates for the related exterior value problems, construct special exterior conditions, and prove the exterior determination results in Sections 5.1, 5.2 and 5.3, respectively. We give a detailed proof of an auxiliary lemma for the used exterior conditions in Appendix A.

## Two inverse conductivity problems

In order to explain the motivations behind our study and the connections it bears with the known literature, in this section we compare the inverse problems for the classical and fractional conductivity equations (see also the surveys [50, 55] for more information and references). This will also serve the purpose of showing the similarities in the mathematical structures of the two problems, the main differences being related to the geometric setting of the measurements and locality/nonlocality questions.

### Two conductivity equations

Let $\Omega \subset {\mathbb {R}}^n$ be a bounded open set, and consider a fixed but unknown conductivity function $\gamma :{\mathbb {R}}^n\rightarrow {\mathbb {R}}^+$. In the classical Calderón problem, which was first introduced in [11], the goal is to recover γ in Ω from measurements of electric potentials and currents *performed on the boundary* $\partial \Omega$ . The electric potential *u* solves the classical conductivity equation

3 $$\begin{aligned} \begin{aligned} {{\,\textrm{div}\,}}(\gamma \nabla u)&=0 \quad \text{ in } \Omega , \\ u&=f \quad \text{ on } \partial \Omega \end{aligned} \end{aligned}$$

for any given boundary value *f* on $\partial \Omega$, and the measurements are given in the form of the DN map $\Lambda _\gamma : H^{1/2}(\partial \Omega )\rightarrow H^{-1/2}(\partial \Omega )$ mapping $f \mapsto \gamma \partial _\nu u|_{\partial \Omega }$.

Let now s∈(0,1). Using the nonlocal vector calculus developed in [20], it is possible to define a fractional conductivity equation [16, 49] as

4 $$\begin{aligned} \begin{aligned} \text{ div}_s (\Theta _\gamma \nabla ^su)&=0 \quad \text{ in } \Omega , \\ u&=f \quad \text{ in } \Omega _e , \end{aligned}\end{aligned}$$

where $\Theta _\gamma$ is an appropriate matrix depending on the conductivity γ, and $\nabla ^s$, $\hbox {div}_s$ are the so called fractional gradient and divergence (see Section 3.1 for the definitions of such operators). The inverse problem for the fractional conductivity equation consists in recovering the conductivity γ from a nonlocal analogue of DN data, which in the case of Lipschitz domains takes the form $\Lambda ^s_\gamma :H^s(\Omega _e)\rightarrow H^{-s}_{\overline{\Omega }_e}({\mathbb {R}}^n)$. We only write here $\Lambda ^s_\gamma$ to distinguish it from the local DN map, but otherwise we suppress the fractional index *s*.

Observe that while the conductivity operator ${{\,\textrm{div}\,}}(\gamma \nabla \cdot )$ is local, the fractional conductivity operator ${{\,\textrm{div}\,}}_s(\Theta _\gamma \nabla ^s\cdot )$ is nonlocal. As the former conserves supports, the values of *u* in $\Omega _e$ do not interfere in the computation of ${{\,\textrm{div}\,}}(\gamma \nabla u)$ in Ω. Thus the classical Dirichlet problem (3) requires just a *boundary value*. On the other hand, this does not suffice for the latter, given that the values of *u* in $\Omega _e$ do interfere in the computation of $\hbox {div}_s(\Theta _\gamma \nabla ^s u)$ in Ω. This is why the fractional Dirichlet problem (4) needs an *exterior value*. These fundamental characteristics also motivate the definitions of the DN maps as shown above, which both naturally derive from the bilinear forms associated with the problems (3) and (4).

### The Liouville reductions

By means of a standard change of variables, the so called *Liouville reduction*, the classical Calderón problem is reduced to an inverse problem for the (time-independent) Schrödinger equation

5 $$\begin{aligned} \begin{aligned} -\Delta u + q_\gamma u&=0 \quad \text{ in } \Omega \\ u&=g \quad \text{ on } \partial \Omega , \end{aligned}\end{aligned}$$

which asks to recover the potential $q_\gamma$ given the relative DN map $\Lambda _{q_\gamma }$. It is then possible to infer results about the classical Calderón problem by studying the inverse problem for (5).

Our previous results [16, 49] show that the described inverse problem for the fractional conductivity equation also allows a *fractional Liouville reduction*, which transforms it into an inverse problem for the fractional Schrödinger equation

6 $$\begin{aligned} \begin{aligned} (-\Delta )^s u + q u&=0 \quad \text{ in } \Omega \\ u&=g \quad \text{ in } \Omega _e , \end{aligned}\end{aligned}$$

where $(-\Delta )^s$ is the fractional Laplacian (see Section 3.1 for the definition). The reduced problem consists in recovering the potential *q* from the nonlocal DN map $\Lambda ^s_q$, whose definition is based on the bilinear form associated to the problem. By analogy to the local case, the inverse problem obtained after the reduction is itself called the fractional Calderón problem. It was introduced in the seminal paper [26], and as such predates the fractional conductivity equation.

### Unique continuation principles and interior uniqueness

Being a prototypical second order elliptic equation, the pioneering work of Carleman [12], see also [2, 5, 33], shows that (5) admits the following strong unique continuation property (SUCP):

*If u* *vanishes of infinite order at* $x_0$, *then* u≡0 *in a neighborhood of* $x_0$.

This is proved using Carleman estimates, which are a special class of weighted $L^2$ estimates. These also allow one to prove the existence of complex geometrical optics (CGO) solutions to (5), which were devised by Sylvester and Uhlmann ( [52, 53]) in order to emulate Calderón’s exponential solutions ( [11]) at high frequencies. Their construction, which was in origin for $C^2$ conductivities for n≥3, has been upgraded for $C^1$ [29], Lipschitz [18] and $W^{1,n}(\Omega )\cap L^\infty (\Omega )$, n=3,4 conductivities [28]. By using such CGO solutions as test functions in an integral identity of the form $\int _{{\mathbb {R}}^n}qu_1u_2 dx=0$ (the so called *Alessandrini identity*), one can prove uniqueness for the classical Calderón problem. In the case n=2, the fundamental result by Astala and Päivärinta [4] proved uniqueness for an $L^\infty (\Omega )$ conductivity using complex analysis techniques. Later on, this result has been improved in certain cases, see for example [9, 41]. Given that the equation does not carry information about γ in $\Omega _e$, all these are *interior uniqueness* results. *Partial data* results are available for n≥3 in specific geometries [22, 30, 32], and in general for n=2 [31].

The situation is quite different in this regard for the fractional Calderón problem. It is in fact known that the fractional Laplacian enjoys the unique continuation property

*Let* $r\in {\mathbb {R}}$ *and* $u \in H^r({\mathbb {R}}^n)$. *If* $u=(-\Delta )^su=0$ *in a nonempty open set, then* u≡0.

Such property of course immediately extends to local perturbations of the fractional Laplacian, such as for the fractional Schrödinger equation (6). It is a classical result when *u* is assumed to be more regular, and was proved for s∈(0,1) in [26] using Carleman estimates from [47] and the Caffarelli–Silvestre extension [19]. This proof was then extended to general $s\in {\mathbb {R}}^+\setminus {\mathbb {N}}$ in [14]. The strength of the unique continuation property stated above for the fractional Calderón problem translates into stronger uniqueness results than in the classical case. Already in [26] uniqueness was proved in all dimensions n≥2 and for partial data in the case of an $L^\infty$ potential. The proof of uniqueness in Ω was extended to global low regularity potentials in $L^{n/2s}({\mathbb {R}}^n)$ and $H^{-s,n/s}({\mathbb {R}}^n)$ in [46]. Even a single measurement was shown to be sufficient for uniqueness in [25]. Many perturbed versions of the fractional Calderón problem have been considered, among which [15, 49] solved the uniqueness question for general local perturbations and [17] for quasilocal perturbations. Our Theorem 1.1 adds to the cited results by proving the interior uniqueness in the inverse problem for the fractional conductivity equation in the case when Ω is open and bounded in one direction and γ is nontrivial in the exterior.

The main technique used in all of these results is quite different from the classical one. Using the fractional unique continuation result one can prove the Runge approximation property [23, 26], which allows one to approximate any sufficiently regular function on Ω by solutions to (6). These are then used as test functions in the Alessandrini identity, thus avoiding the need for CGO solutions altogether.

### Exterior and boundary determination

The recovery of a smooth conductivity γ up to infinite order on the boundary $\partial \Omega$ has been established by Kohn and Vogelius in [34] and as an application they obtained the global recovery of piecewise real analytic conductivities [35]. These results were later extended to continuous conductivities [8, 54]. Our Theorem 1.2 serves the purpose of positively answering the very natural question of whether in the fractional Calderón problem γ can be uniquely recovered in $\Omega _e$. By analogy to the boundary determination question studied by Kohn and Vogelius, we call such problem *exterior determination*. Theorem 1.3 gives conditions under which the global uniqueness can be achieved.

### Stability and reconstruction questions

In [3], Alessandrini proved that in dimension n≥3 there exists a logarithmic modulus of continuity

$$\begin{aligned} \Vert \gamma _1-\gamma _2\Vert _{L^\infty (\Omega )} \le C \left( |\log \Vert \Lambda _1-\Lambda _2\Vert _{\frac{1}{2},-\frac{1}{2}}|^{-\sigma }+ \Vert \Lambda _1-\Lambda _2\Vert _{\frac{1}{2},-\frac{1}{2}} \right) \;,\quad \sigma \in (0,1) \end{aligned}$$

for smooth conductivities in the case of the classical Calderón problem. Such estimate was then showed to be optimal by Mandache [38]. This implies that the classical Calderón problem is severely ill-posed. However, Nachman and Novikov were able to show a reconstruction result for the Schrödinger and conductivity equations [39, 40] from the associated DN map using CGO techniques.

The fractional Calderón problem was shown to be severely ill-posed as well [43–46]. In fact, since the following (optimal) estimate holds

$$\begin{aligned} \Vert q_1-q_2\Vert _{L^{n/2s}(\Omega )} \le C |\log \Vert \Lambda _{q_1}-\Lambda _{q_2}\Vert _* |^{-\sigma }\;,\quad \sigma \in (0,1)\;, \end{aligned}$$

the fractional Calderón problem enjoys only logarithmic stability. In Proposition 1.4, we show that Lipschitz stability *in the exterior* can be achieved for the fractional conductivity equation. Interior reconstruction of a low regularity potential was shown to be possible even in the case of a single measurement in [25], and the result extends to the fractional conductivity equation by means of the fractional Liouville reduction for conductivities which are known to be trivial in the *whole* exterior [16]. Our Lemma 5.7 gives a novel *exterior* reconstruction result, which is reminiscent of the discussed boundary determination by Nachman and Novikov for the classical Calderón problem.

## Preliminaries

### Bessel potential spaces, fractional Laplacians and fractional gradients

We denote the space of Schwartz functions by $\mathscr {S}({\mathbb {R}}^n)$ and its dual space, the space of tempered distributions, by $\mathscr {S}^{\prime }({\mathbb {R}}^n)$. The Fourier transform of $u\in \mathscr {S}({\mathbb {R}}^n)$ is defined as

$$ \mathcal {F}u(\xi ):= \hat{u}(\xi ) := \int _{{\mathbb {R}}^n} u(x)e^{-ix \cdot \xi } \,dx. $$

$\mathcal {F}$ acts as an isomorphism on $\mathscr {S}({\mathbb {R}}^n)$, and by duality on $\mathscr {S}^{\prime }({\mathbb {R}}^n)$ as well. We denote the inverse of the Fourier transform by $\mathcal {F}^{-1}$. The Bessel potential of order $s \in {\mathbb {R}}$ is the Fourier multiplier $\left\langle D\right\rangle ^s:\mathscr {S}^{\prime }({\mathbb {R}}^n) \rightarrow \mathscr {S}^{\prime }({\mathbb {R}}^n)$, that is

$$\begin{aligned} \left\langle D\right\rangle ^s u := \mathcal {F}^{-1}(\left\langle \xi \right\rangle ^s\widehat{u}), \end{aligned}$$

where $\left\langle \xi \right\rangle := (1+|\xi |^2)^{1/2}$. If $s \in {\mathbb {R}}$ and $1 \le p \le \infty$, the Bessel potential space $H^{s,p}({\mathbb {R}}^n)$ is

$$\begin{aligned} H^{s,p}({\mathbb {R}}^n) := \{ u \in \mathscr {S}^{\prime }({\mathbb {R}}^n)\,;\, \left\langle D\right\rangle ^su \in L^p({\mathbb {R}}^n)\}, \end{aligned}$$

endowed with the norm $\Vert u \Vert _{H^{s,p}({\mathbb {R}}^n)} := \Vert \left\langle D\right\rangle ^su \Vert _{L^p({\mathbb {R}}^n)}$. Given an open set $\Omega \subset {\mathbb {R}}^n$, we define the following local Bessel potential space:

$$\begin{aligned}\begin{aligned} \widetilde{H}^{s,p}(\Omega )&:= \text{ closure } \text{ of } C_c^\infty (\Omega ) \text{ in } H^{s,p}({\mathbb {R}}^n). \end{aligned} \end{aligned}$$

We see that $\widetilde{H}^{s,p}(\Omega )$ is a closed subspace of $H^{s,p}({\mathbb {R}}^n)$. As customary, we omit the index *p* from the above notations in the case p=2. We shall also make use of the equivalent *Gagliardo–Slobodeckij norms*

7 $$\begin{aligned} \Vert u \Vert _{W^s({\mathbb {R}}^n)} := \sqrt{\Vert u \Vert _{L^2({\mathbb {R}}^n)}^2+\Vert (-\Delta )^{s/2}u \Vert _{L^2({\mathbb {R}}^n)}^2} \end{aligned}$$

for $u \in H^s({\mathbb {R}}^n)$. We remark that in some references the norms may be different by a constant appearing in the second term $\Vert (-\Delta )^{s/2}u \Vert _{L^2({\mathbb {R}}^n)}$.

If $u\in \mathscr {S}^{\prime }({\mathbb {R}}^n)$ is a tempered distribution and s≥0, the fractional Laplacian of order *s* of *u* is the Fourier multiplier

$$ (-\Delta )^su:= \mathcal {F}^{-1}(|\xi |^{2s}\widehat{u}), $$

whenever the right hand side is well-defined. If p≥1 and $t\in {\mathbb {R}}$, the fractional Laplacian is a bounded linear operator $(-\Delta )^{s}:H^{t,p}({\mathbb {R}}^n) \rightarrow H^{t-2s,p}({\mathbb {R}}^n)$. In the special case $u\in \mathscr {S}({\mathbb {R}}^n)$ and s∈(0,1), we have the identities (see e.g. [21, Section 3])

$$\begin{aligned} \begin{aligned} (-\Delta )^su(x)&=C_{n,s}\,\text {p.v.}\int _{{\mathbb {R}}^n}\frac{u(x)-u(y)}{|x-y|^{n+2s}}\,dy\\&= -\frac{C_{n,s}}{2}\int _{{\mathbb {R}}^n}\frac{u(x+y)+u(x-y)-2u(x)}{|y|^{n+2s}}\,dy, \end{aligned} \end{aligned}$$

where $C_{n,s}:=\frac{4^s \Gamma (n/2+s)}{\pi ^{n/2}|\Gamma (-s)|}.$ Here $\text{ p.v. }$ indicates the Cauchy principal value; it is needed in the former integral, which is singular, but can be omitted in the latter, as observed by [21].

Let now s∈(0,1). The fractional gradient of order *s* is the bounded linear operator $\nabla ^s:H^s({\mathbb {R}}^n)\rightarrow L^2({\mathbb {R}}^{2n};{\mathbb {R}}^n)$ given by (see [16, 20, 49] and [21, Propositions 3.4 and 3.6])

$$ \nabla ^su(x,y):=\sqrt{\frac{C_{n,s}}{2}}\frac{u(x)-u(y)}{|x-y|^{n/2+s+1}}(x-y), $$

with

$$\begin{aligned} \Vert \nabla ^su\Vert _{L^2({\mathbb {R}}^{2n})}=\Vert (-\Delta )^{s/2}u\Vert _{L^2({\mathbb {R}}^n)}\le \Vert u\Vert _{H^s({\mathbb {R}}^n)}. \end{aligned}$$

The fractional divergence of order *s* is the bounded linear operator

$$\begin{aligned} {{\,\textrm{div}\,}}_s:L^2({\mathbb {R}}^{2n};{\mathbb {R}}^n)\rightarrow H^{-s}({\mathbb {R}}^n) \end{aligned}$$

given by

$$ \langle {{\,\textrm{div}\,}}_su,v\rangle _{H^{-s}({\mathbb {R}}^n)\times H^s({\mathbb {R}}^n)}=\langle u,\nabla ^sv\rangle _{L^2({\mathbb {R}}^{2n})} $$

for all $u\in L^2({\mathbb {R}}^{2n};{\mathbb {R}}^n),v\in H^s({\mathbb {R}}^n)$, that is, ${{\,\textrm{div}\,}}_s$ is by definition the adjoint of $\nabla ^s$. One can show that (see [49, Section 8])

$$ \Vert {{\,\textrm{div}\,}}_s(u)\Vert _{H^{-s}({\mathbb {R}}^n)}\le \Vert u\Vert _{L^2({\mathbb {R}}^{2n})} $$

for all $u\in L^2({\mathbb {R}}^{2n};{\mathbb {R}}^n)$, and also $(-\Delta )^su={{\,\textrm{div}\,}}_s(\nabla ^su)$ weakly for all $u\in H^s({\mathbb {R}}^n)$ (see [16, Lemma 2.1]).

One also has the following product rule for the fractional gradient

8 $$\begin{aligned} \begin{aligned}&\nabla ^s(\phi \psi )(x,y)=\sqrt{\frac{C_{n,s}}{2}}\frac{x-y}{|x-y|^{n/2+s+1}}(\phi (x)\psi (x)-\phi (y)\psi (y))\\&=\sqrt{\frac{C_{n,s}}{2}}\frac{x-y}{|x-y|^{n/2+s+1}}(\phi (x)\psi (x)-\phi (x)\psi (y)+\phi (x)\psi (y)-\phi (y)\psi (y))\\&=\phi (x)\nabla ^s\psi (x,y)+\psi (y)\nabla ^s\phi (x,y) \end{aligned} \end{aligned}$$

for all $\phi ,\psi \in H^s({\mathbb {R}}^n)$ and a.e. $x,y\in {\mathbb {R}}^n$.

### Domains bounded in one direction and fractional Poincaré inequalities

#### Definition 3.1

Let $n,k\in {\mathbb {N}}$ with 0<k≤n, and assume $\omega \subset {\mathbb {R}}^k$ is a bounded open set. The set $\Omega _{\infty }:={\mathbb {R}}^{n-k}\times \omega \subset {\mathbb {R}}^n$ is a *cylindrical domain*.

Let $n\in {\mathbb {N}}$ and assume $\Omega \subset {\mathbb {R}}^n$ is an open set. If there exist a cylindrical domain $\Omega _{\infty } \subset {\mathbb {R}}^n$ and a rigid Euclidean motion *A* such that $\Omega \subset A(\Omega _{\infty })$, we say that Ω is *bounded in one direction*.

#### Theorem 3.2

(Poincaré inequality (cf. [49, Theorem 2.2])) Let $\Omega \subset {\mathbb {R}}^n$ be an open set that is bounded in one direction. Suppose that $2 \le p < \infty$ and $0\le s\le t < \infty$, or 1<p<2, $1 \le t < \infty$ and $0 \le s \le t$. Then there exists C(n,p,s,t,Ω)>0 such that

$$\begin{aligned} \Vert (-\Delta )^{s/2}u\Vert _{L^p({\mathbb {R}}^n)}\le C\Vert (-\Delta )^{t/2}u\Vert _{L^p({\mathbb {R}}^n)} \end{aligned}$$

for all $$u\in \widetilde{H}^{t,p}(\Omega )$$.

### Bilinear forms and DN maps for the fractional conductivity and Schrödinger equations

Let us briefly define the space of bounded Sobolev multipliers in $H^{s}({\mathbb {R}}^n)$. Let $f\in \mathscr {D}^{\prime }({\mathbb {R}}^n)$ be a distribution. Then we say that $f\in M(H^r\rightarrow H^t)$ whenever the norm

$$\begin{aligned} \Vert f\Vert _{r,t} := \sup \{\left\lvert \left\langle f,u v \right\rangle \right\rvert \,;\, u,v \in C_c^\infty (\mathbb {R}^n), \Vert u \Vert _{H^r({\mathbb {R}}^n)} = \Vert v \Vert _{H^{-t}({\mathbb {R}}^n)} =1 \} \end{aligned}$$

is finite. The space $M_0(H^r \rightarrow H^t)$ is defined as the closure of $C_c^\infty ({\mathbb {R}}^n)$ in $M(H^r \rightarrow H^t)$.

#### Lemma 3.3

(Definition of bilinear forms and conductivity matrix (cf. [49, Lemma 8.3])) Let 0<s<min(1,n/2), $q\in L^{\frac{n}{2s}}({\mathbb {R}}^n)$, $\gamma \in L^{\infty }({\mathbb {R}}^n)$ and define the conductivity matrix associated to γ by

$$\begin{aligned} \Theta _{\gamma }:{\mathbb {R}}^{2n}\rightarrow {\mathbb {R}}^{n\times n},\quad \Theta _{\gamma }(x,y):=\gamma ^{1/2}(x)\gamma ^{1/2}(y)\textbf{1}_{n\times n} \end{aligned}$$

for $x,y\in {\mathbb {R}}^n$. Then the maps $B_{\gamma }:H^s({\mathbb {R}}^n)\times H^s({\mathbb {R}}^n) \rightarrow {\mathbb {R}}$ and $B_q:H^s({\mathbb {R}}^n)\times H^s({\mathbb {R}}^n)\rightarrow {\mathbb {R}}$ defined by

$$\begin{aligned} B_{\gamma }(u,v):=\int _{{\mathbb {R}}^{2n}}\Theta _{\gamma }\nabla ^su\cdot \nabla ^sv\,dxdy \end{aligned}$$

and

$$\begin{aligned} \quad B_q(u,v):=\int _{{\mathbb {R}}^n}(-\Delta )^{s/2}u\,(-\Delta )^{s/2}v\,dx+\int _{{\mathbb {R}}^n}quv\,dx \end{aligned}$$

are continuous bilinear forms. Moreover, we have $q\in M_0(H^s\rightarrow H^{-s})$ with

$$\begin{aligned} \Vert q\Vert _{s,-s}\le C(1+\Vert q\Vert _{L^{\frac{n}{2s}}({\mathbb {R}}^n)}) \end{aligned}$$

for some C>0.

#### Definition 3.4

*(Weak solutions)* Let $\Omega \subset {\mathbb {R}}^n$ be an open set, 0<s<min(1,n/2), $q\in L^{\frac{n}{2s}}({\mathbb {R}}^n)$ and $\gamma \in L^{\infty }({\mathbb {R}}^n)$ with conductivity matrix $\Theta _\gamma :{\mathbb {R}}^{2n}\rightarrow {\mathbb {R}}^{n\times n}$. If $f\in H^s({\mathbb {R}}^n)$ and $F\in (\widetilde{H}^s(\Omega ))^*$, then we say that $u\in H^s({\mathbb {R}}^n)$ is a weak solution to the fractional conductivity equation

$$ \begin{aligned} {{\,\textrm{div}\,}}_s(\Theta _\gamma \nabla ^s u)&= F\quad \text {in}\quad \Omega ,\\ u&= f\quad \text {in}\quad \Omega _e, \end{aligned} $$

if there holds

$$ B_{\gamma }(u,\phi )=F(\phi )\quad \text {and}\quad u-f\in \widetilde{H}^s(\Omega ) $$

for all $$\phi \in \widetilde{H}^s(\Omega )$$.

Similarly, we say that $v\in H^s({\mathbb {R}}^n)$ is a weak solution to the fractional Schrödinger equation

$$ \begin{aligned} ((-\Delta )^s+q)v&=F\quad \text {in}\quad \Omega ,\\ v&=f\quad \text {in}\quad \Omega _e, \end{aligned} $$

if there holds

$$ B_q(v,\phi )=F(\phi )\quad \text {and}\quad v-f\in \widetilde{H}^s(\Omega ) $$

for all $$\phi \in \widetilde{H}^s(\Omega )$$.

By standard arguments and the fractional Poincaré inequality on domains bounded in one direction (Theorem 3.2) one can show the following result:

#### Lemma 3.5

(Well-posedness and DN maps (cf. [49, Lemma 8.10])) Let $\Omega \subset {\mathbb {R}}^n$ be an open set which is bounded in one direction and 0<s<min(1,n/2). Assume that $\gamma \in L^{\infty }({\mathbb {R}}^n)$ with conductivity matrix $\Theta _{\gamma }$, background deviation $m_{\gamma }$ and electric potential $q_{\gamma }:= -\frac{(-\Delta )^sm_{\gamma }}{\gamma ^{1/2}}$ satisfies $\gamma (x)\ge \gamma _0>0$ and $m_{\gamma }\in H^{2s,\frac{n}{2s}}({\mathbb {R}}^n)$. Then the following assertions hold:

(i) For all $f\in X:= H^s({\mathbb {R}}^n)/\widetilde{H}^s(\Omega )$ there are unique weak solutions $u_f,v_f\in H^s({\mathbb {R}}^n)$ to the fractional conductivity equation
  9 $$\begin{aligned} \begin{aligned} {{\,\textrm{div}\,}}_s(\Theta _{\gamma }\nabla ^s u)&= 0\quad \text {in}\quad \Omega ,\\ u&= f\quad \text {in}\quad \Omega _e \end{aligned} \end{aligned}$$
  and to the fractional Schrödinger equation
  $$\begin{aligned} {\begin{matrix} ((-\Delta )^s+q_{\gamma })v& =0\quad \text {in}\quad \Omega ,\\ v& =f\quad \text {in}\quad \Omega _e. \end{matrix}} \end{aligned}$$
(ii) The exterior DN maps $\Lambda _{\gamma }:X\rightarrow X^*$, $\Lambda _{q_{\gamma }}:X\rightarrow X^*$ given by
  10 $$\begin{aligned} \begin{aligned} \langle \Lambda _{\gamma }f,g\rangle :=B_{\gamma }(u_f,g),\quad \langle \Lambda _{q_\gamma }f,g\rangle :=B_{q_{\gamma }}(v_f,g), \end{aligned} \end{aligned}$$
  are well-defined bounded linear maps.

#### Remark 3.6

If no ambiguities arise, we will drop later in this article the subscripts attached to the conductivity matrix, the background deviation and the electric potential.

## Unique continuation property of exterior DN maps and invariance of data

We give the proof of Theorem 1.1 in this section. Theorem 1.1 is based on the works [16, 19, 26, 45, 47, 49], multiplication results in Bessel potential spaces (see e.g. [1, 7, 45, 49]) and different equivalent characterizations of fractional Laplacians (see e.g. [21, 36, 51]). The strategy of our proof is the following:

(i) We first define the fractional Liouville reduction given in Lemmas 3.5 and 4.2, which transforms the exterior DN maps of the fractional conductivity equation to the exterior DN maps of the fractional Schrödinger equation with the global singular potentials $q_\gamma = -\frac{(-\Delta )^s (\gamma ^{1/2}-1)}{\gamma ^{1/2}}$. This transformation was first introduced in [16] and further generalized for global low regularity conductivities in [49]. One uses the formula
  $$\begin{aligned} (-\Delta )^su(x)=-\frac{C_{n,s}}{2}\int _{{\mathbb {R}}^n}\frac{u(x+y)+u(x-y)-2u(x)}{|y|^{n+2s}}\,dy, \end{aligned}$$
  $\hbox {weak}^*$ approximation of conductivities, and multiplication results of Bessel potential spaces in the proof of the Liouville reduction.
(ii) It follows that if $\gamma _1|_{W_1 \cup W_2} = \gamma _2|_{W_1 \cup W_2}$ and $\Lambda _{\gamma _1}f|_{W_2} = \Lambda _{\gamma _2}f|_{W_2}$ for all $f \in C_c^\infty (W_1)$, then $\Lambda _{q_1}f|_{W_2} = \Lambda _{q_2}f|_{W_2}$. This is based on a modification of the arguments for some simpler special cases considered in [16, 49].
(iii) Now one may invoke the interior uniqueness result of the fractional Calderón problem for globally defined singular potentials [45]: If $\Lambda _{q_1}f|_{W_2} = \Lambda _{q_2}f|_{W_2}$ for all $f \in C_c^\infty (W_1)$, then $q_1 = q_2$ in Ω. This is based on the UCP of the fractional Laplacian and the Runge approximation argument in [26].
(iv) Next we may use the exterior determination argument of [49], which states that if $\Lambda _{q_1}f|_{W_2} = \Lambda _{q_2}f|_{W_2}$ for all $f \in C_c^\infty (W_1)$ and $W=W_1\cap W_2 \ne \emptyset$, then $q_1=q_2$ in *W*. This uses the earlier interior determination step, which already guarantees that $q_1 = q_2$ in Ω.
(v) By the assumption that $\gamma _1|_W = \gamma _2|_W$ and the knowledge of
  $$\begin{aligned}-\frac{(-\Delta )^s (\gamma _1^{1/2}-1)}{\gamma _1^{1/2}} = q_1 = q_2 = -\frac{(-\Delta )^s (\gamma _2^{1/2}-1)}{\gamma _2^{1/2}}\quad \text {in { W}} \end{aligned}$$
  together with the UCP of the fractional Laplacian, we can conclude that $\gamma _1 = \gamma _2$ in ${\mathbb {R}}^n$, as desired.
(vi) The extension to domains bounded in one direction is based on the theory developed in [49]. The used low regularity assumptions are found by considering multiplication results of Bessel potentials spaces and the basic mapping properties of the fractional Laplacian.

### Remark 4.1

In step (iii) it is important to use data $\left\langle \Lambda _{q_1}f,g \right\rangle = \left\langle \Lambda _{q_2}f,g \right\rangle$ such that ${{\,\textrm{supp}\,}}(f)\cap {{\,\textrm{supp}\,}}(g) = \emptyset$. In step (iv) the data must instead verify ${{\,\textrm{supp}\,}}(f)\cap {{\,\textrm{supp}\,}}(g)\ne \emptyset$.

The proof of Theorem 1.1 (ii) uses the same arguments up to point (iii). The argument after that differ slightly, and are mainly of interest for the counterexamples which were already discussed in the Introduction.

### Proof of Theorem 1.1

We begin with a simple lemma related to the fractional Liouville reduction.

#### Lemma 4.2

Let $\Omega \subset {\mathbb {R}}^n$ be an open set which is bounded in one direction, $W\subset \Omega _e$ an open set and 0<s<min(1,n/2). Assume that $\gamma ,\Gamma \in L^{\infty }({\mathbb {R}}^n)$ with background deviations $m_{\gamma },m_{\Gamma }$ satisfy $\gamma (x),\Gamma (x)\ge \gamma _0>0$ and $m_{\gamma },m_{\Gamma }\in H^{2s,\frac{n}{2s}}({\mathbb {R}}^n)$. If $\gamma |_{W}=\Gamma |_{W}$, then

$$ \langle \Lambda _{\gamma }f,g\rangle =\langle \Lambda _{q_\gamma }(\Gamma ^{1/2}f),(\Gamma ^{1/2}g)\rangle $$

holds for all $f,g\in \widetilde{H}^{s}(W)$.

#### Proof

Observe that any $f\in \widetilde{H}^s(W)$ corresponds to a unique element of *X*. Hence the regularity assumptions on Γ and [49, Corollary A.8] imply that $\Gamma ^{1/2}f\in \widetilde{H}^{s}(W)$ for any $f\in \widetilde{H}^s(W)$, which makes the DN maps in (10) well-defined. If $u_f\in H^s({\mathbb {R}}^n)$ is the unique solution to

$$ \begin{aligned} {{\,\textrm{div}\,}}_s(\Theta _\gamma \nabla ^s u)&= 0\quad \text {in}\quad \Omega ,\\ u&= f\quad \text {in}\quad \Omega _e \end{aligned} $$

with $f\in \widetilde{H}^s(W)$, by the fractional Liouville reduction (see [49, Theorem 8.6]) $\gamma ^{1/2}u_f\in H^s({\mathbb {R}}^n)$ solves

$$ \begin{aligned} ((-\Delta )^s+q_\gamma )v&=0\quad \quad \,\,\,\,\,\text {in}\quad \Omega ,\\ v&=\gamma ^{1/2}f\quad \text {in}\quad \Omega _e. \end{aligned} $$

Since $\gamma |_W = \Gamma |_W$, we have that $\gamma ^{1/2}u_f$ is the unique solution to

$$ \begin{aligned} ((-\Delta )^s+q_\gamma )v&=0\quad \quad \,\,\,\,\,\text {in}\quad \Omega ,\\ v&=\Gamma ^{1/2}f\quad \text {in}\quad \Omega _e, \end{aligned} $$

which we denote by $v_{\Gamma ^{1/2}f}$.

Therefore, by [49, Remark 8.8], we get

$$ \begin{aligned} \langle \Lambda _{\gamma }f,g\rangle&=B_{\gamma }(u_f,g)=B_{q_\gamma }(\gamma ^{1/2}u_f,\gamma ^{1/2}g)=B_{q_\gamma }(v_{\Gamma ^{1/2}f},\Gamma ^{1/2}g)\\&=\langle \Lambda _{q_\gamma }(\Gamma ^{1/2}f),(\Gamma ^{1/2}g)\rangle \end{aligned} $$

for all $$f,g\in \widetilde{H}^s(W)$$. □

We can now prove Theorem 1.1, which generalizes [49, Lemma 8.15]. Here we focus mainly on the differences in the arguments which allow the generalization, and refer to the cited result for the missing details.

#### Proof of Theorem 1.1

Throughout the proof let $\Gamma \in L^{\infty }({\mathbb {R}}^n)$ be any function satisfying $\Gamma \ge \gamma _0$, $m_{\Gamma }:=\Gamma ^{1/2}-1\in H^{2s,\frac{n}{2s}}({\mathbb {R}}^n)$ and $\Gamma =\gamma _1=\gamma _2$ in $W_1\cup W_2$. Notice that there holds

$$ \langle \Lambda _{\gamma _1}f,g\rangle =\langle \Lambda _{\gamma _2}f,g\rangle $$

for all $f\in \widetilde{H}^s(W_1)$, $g\in \widetilde{H}^{s}(W_2)$ if and only if we have

$$ \langle \Lambda _{\gamma _1}f,g\rangle =\langle \Lambda _{\gamma _2}f,g\rangle $$

for all $f\in C_c^{\infty }(W_1)$, $g\in C_c^{\infty }(W_2)$. This follows by approximation and the continuity of $\Lambda _{\gamma _j}: X \rightarrow X^*$ for j=1,2 (see Lemma 3.5 (ii)). Similarly, one can show that there holds

$$ \langle \Lambda _{q_1}f,g\rangle =\langle \Lambda _{q_2}f,g\rangle $$

for all $f\in C_c^{\infty }(W_1)$, $g\in C_c^{\infty }(W_2)$ if and only if

$$ \langle \Lambda _{q_1}f,g\rangle =\langle \Lambda _{q_2}f,g\rangle $$

for all $$f\in \widetilde{H}^s(W_1)$$, $$g\in \widetilde{H}^{s}(W_2)$$.

Now, by the first part of the proof and Lemma 4.2, the condition $\Lambda _{\gamma _1}f|_{W_2}=\Lambda _{\gamma _2}f|_{W_2}$ for all $f\in C_c^{\infty }(W_1)$ is equivalent to

11 $$\begin{aligned} \langle \Lambda _{q_1}(\Gamma ^{1/2}f),(\Gamma ^{1/2}g)\rangle =\langle \Lambda _{q_2}(\Gamma ^{1/2}f),(\Gamma ^{1/2}g)\rangle \end{aligned}$$

for all $f\in \widetilde{H}^s(W_1)$, $g\in \widetilde{H}^s(W_2)$. Next note that we can write

$$ \frac{1}{\Gamma ^{1/2}}=1-\frac{m_{\Gamma }}{m_{\Gamma }+1} $$

and set $\Gamma _0:=\min (0,\gamma _0^{1/2}-1)$. Let $H\in C^2_b({\mathbb {R}})$ satisfy $H(t)=\frac{t}{t+1}$ for $t\ge \Gamma _0$. By [1, p. 156] and $m_{\Gamma }\in H^{2s,\frac{n}{2s}}({\mathbb {R}}^n)\cap L^{\infty }({\mathbb {R}}^n)$, we deduce $H(m_{\Gamma })\in H^{2s,\frac{n}{2s}}({\mathbb {R}}^n)$, but since $m_{\Gamma }\ge \gamma _0^{1/2}-1$ it follows that $\frac{m_{\Gamma }}{m_{\Gamma }+1}\in H^{2s,\frac{n}{2s}}({\mathbb {R}}^n)\cap L^{\infty }({\mathbb {R}}^n)$. Therefore, by [49, Corollary A.8] we see that (11) is equivalent to

$$\begin{aligned} \langle \Lambda _{q_1}f,g\rangle =\langle \Lambda _{q_2}f,g\rangle \end{aligned}$$

for all $$f\in \widetilde{H}^s(W_1)$$, $$g\in \widetilde{H}^s(W_2)$$.

Now we can follow the proof of Lemma 8.15 in [49] to conclude that this is equivalent to the assertion that $m_1-m_2\in H^s({\mathbb {R}}^n)$ is the unique solution of

$$\begin{aligned} \begin{aligned} (-\Delta )^sm-\frac{(-\Delta )^sm_1}{\gamma _1^{1/2}}m&=0\quad \text {in}\quad \Omega ,\\ m&=m_0\quad \text {in}\quad \Omega _e, \end{aligned} \end{aligned}$$

and

12 $$\begin{aligned} \int _{\Omega _e}\frac{(-\Delta )^sm_0}{\Gamma ^{1/2}}fg\,dx=0 \end{aligned}$$

for all $$f\in C^{\infty }_c(W_1)$$, $$g\in C_c^{\infty }(W_2)$$.

If the sets $W_1,W_2\subset \Omega _e$ are disjoint, we immediately obtain the statement (ii). Assume now that $W_1\cap W_2\ne \emptyset$, and let $\Gamma ^{1/2}_{\epsilon }:= \Gamma ^{1/2}*\rho _{\epsilon }$, where $\rho _{\epsilon }$ is the standard mollifier. By standard arguments, one deduces $\Gamma _{\epsilon }^{1/2}\in C^{\infty }_b({\mathbb {R}}^n)$ and $\Gamma _{\epsilon }^{1/2}\overset{*}{\rightharpoonup }\ \Gamma ^{1/2}$. Therefore, by taking $g:= \Gamma _{\epsilon }^{1/2}h\in C^{\infty }_c(W_2)$ with $h\in C_c^{\infty }(W_2)$ in (12) we see that there holds

$$ \int _{\Omega _e}\left( \frac{(-\Delta )^sm_0}{\Gamma ^{1/2}}fh\right) \Gamma _{\epsilon }^{1/2}\,dx=0 $$

for all $f\in C_c^{\infty }(W_1)$, $h\in C_c^{\infty }(W_1)$. Since the expression in the brackets belongs to $L^1({\mathbb {R}}^n)$ and $\Gamma _{\epsilon }^{1/2}\overset{*}{\rightharpoonup }\ \Gamma ^{1/2}$ in $L^{\infty }({\mathbb {R}}^n)$, we obtain in the limit $\epsilon \rightarrow 0$ the identity

$$ \int _{\Omega _e}(-\Delta )^sm_0fh\,dx=0 $$

for all $$f\in C_c^{\infty }(W_1)$$, $$h\in C_c^{\infty }(W_1)$$.

As $W_1\cap W_2\ne \emptyset$, we can choose an open bounded set $\omega \subset {\mathbb {R}}^n$ such that $\overline{\omega }\subset W_1\cap W_2$ and a cutoff function $f\in C_c^{\infty }(W_1)$ such that $f|_{\overline{\omega }}=1$. This gives

$$ \int _{\omega }(-\Delta )^sm_0g\,dx=0 $$

for all $g\in C_c^{\infty }(\omega )$, which in turn implies $(-\Delta )^sm_0=0$ in ω. On the other hand, we have by the assumption $\gamma _1=\gamma _2$ in ω and hence the UCP (cf. [26, Theorem 1.2]) implies $m_0\equiv 0$. This shows $\gamma _1=\gamma _2$, which concludes the proof of (i). □

#### Remark 4.3

Note that by definition of the dual map and [49, Corollary A.11] the second assertion is equivalent to

$$ \langle ((-\Delta )^sm_0)f,\Gamma ^{-1/2}g\rangle _{H^{-s}({\mathbb {R}}^n)\times H^s({\mathbb {R}}^n)}=0 $$

for all $f\in C^{\infty }_c(W_1)$, $g\in C_c^{\infty }(W_2)$. Now using $\Gamma ^{-1/2}=1-\frac{m_{\Gamma }}{m_{\Gamma +1}}$ with $\frac{m_{\Gamma }}{m_{\Gamma +1}}\in H^{2s,\frac{n}{2s}}({\mathbb {R}}^n)\cap L^{\infty }({\mathbb {R}}^n)$, [49, Corollary A.8] and [49, Corollary A.11] we see that this is equivalent to

$$ \langle ((-\Delta )^sm_0)f,\Gamma ^{-1/2}g\rangle _{H^{-s}({\mathbb {R}}^n)\times H^s({\mathbb {R}}^n)}=0 $$

$f\in \widetilde{H}^s(W_1)$, $g\in \widetilde{H}^{s}(W_2)$. Applying once more [49, Corollary A.8] we deduce that this is the same as

$$ \langle ((-\Delta )^sm_0)f,g\rangle _{H^{-s}({\mathbb {R}}^n)\times H^s({\mathbb {R}}^n)}=0 $$

for all $f\in \widetilde{H}^s(W_1)$, $g\in \widetilde{H}^{s}(W_2)$. This directly shows that $(-\Delta )^sm_0$ vanishes as a distribution on $W_1\cap W_2$.

#### Remark 4.4

Suppose that 1/4<s<1. Observe that if the assumptions $\gamma _1=\gamma _2$ in some measurable set $A\subset W_1\cap W_2$ with positive measure, $m_0:= m_1-m_2\in H^r({\mathbb {R}}^n)$ for some $r\in {\mathbb {R}}$ and $\Lambda _{\gamma _1}f|_{W_2}=\Lambda _{\gamma _2}f|_{W_2}$ for all $f\in C_c^{\infty }(W_1)$ still implied that $(-\Delta )^sm_0=0$ in some open set $V\subset W_1\cap W_2$ containing *A*, then using [25, Theorem 3, Remark 5.6] we could conclude that there holds $\gamma _1=\gamma _2$ in ${\mathbb {R}}^n$. The main obstruction in carrying out this analysis is that in this setting we only have the identity

$$ \langle \Lambda _{q_1}(\gamma _1^{1/2}f), (\gamma ^{1/2}_1g)\rangle =\langle \Lambda _{q_2}(\gamma _2^{1/2}f), (\gamma ^{1/2}_2g)\rangle $$

for all $$f\in \widetilde{H}^s(W_1)$$, $$g\in \widetilde{H}^s(W_2)$$.

## Exterior determination for the fractional conductivity equation

We prove in this section our exterior determination results. We begin by proving $H^s \lesssim L^2$ energy estimates for the related exterior value problems in Section 5.1. We then construct sequences of exterior conditions such that the fractional energies can be localized in the limit to any fixed point in ${\mathbb {R}}^n$. Finally, we combine these two important steps to obtain the exterior determination results in the end of this section. In particular, our strategy is the following:

(i) Define the Dirichlet energy first as
  $$\begin{aligned}E_{\gamma }(u):= B_{\gamma }(u,u)= \int _{{\mathbb {R}}^{2n}}\Theta _{\gamma }\nabla ^su\cdot \nabla ^su\,dxdy. \end{aligned}$$
  Notice then that it must be $E_\gamma (u_f) = \left\langle \Lambda _\gamma f,f \right\rangle _{X^*\times X}$, where $u_f$ is the unique solution of the fractional conductivity equation with the exterior condition *f*.
(ii) We first show an elliptic energy estimate for the solutions of the fractional Schrödinger equations (see Lemma 5.3): Let $W \subset \Omega _e$. If $f\in C_c^{\infty }(W)$ and $u_f\in H^s({\mathbb {R}}^n)$ is the unique solution of
  $$\begin{aligned} \begin{aligned} ((-\Delta )^s+q)u&= 0\quad \text {in}\quad \Omega ,\\ u&= f\quad \text {in}\quad \Omega _e, \end{aligned} \end{aligned}$$
  then
  $$\begin{aligned} \Vert u_f-f\Vert _{H^s({\mathbb {R}}^n)}\le C\Vert f\Vert _{L^2(W)} \end{aligned}$$
  for some $C(n,s,|W|,\Omega ,{{\,\textrm{dist}\,}}(W,\Omega ))>0$. This uses the quadratic definition of the fractional Laplacian
  $$\begin{aligned} \left\langle (-\Delta )^sf,\phi \right\rangle =\frac{C_{n,s}}{2}\int _{{\mathbb {R}}^{2n}}\frac{(f(x)-f(y))(\phi (x)-\phi (y))}{|x-y|^{n+2s}}dxdy. \end{aligned}$$
(iii) We then extend this elliptic energy estimate to the fractional conductivity equation (1) in Corollary 5.4 by means of the fractional Liouville reduction.
(iv) We consider sequences of exterior conditions $\phi _N \in C_c^\infty (W)$ such that $\Vert \phi _N \Vert _{L^2(W)} \rightarrow 0$ as $N\rightarrow \infty$ and $\Vert \phi _N \Vert _{H^s({\mathbb {R}}^n)}=1$ for all $N \in {\mathbb {N}}$. Let $u_N \in H^s({\mathbb {R}}^n)$ be the unique solution to the conductivity equation (1) with $u_N|_{\Omega _e} = \phi _N$. The elliptic energy estimate of (iii) and the given properties of the exterior conditions already guarantee that the Dirichlet energies $E_\gamma (u_N)$ and $E_\gamma (\phi _N)$ are asymptotically equivalent as $N \rightarrow \infty$. These exterior conditions are given in Lemma 5.5 and are similar to the boundary conditions considered by Kohn and Vogelius in [34].
(v) Given any $x_0 \in W$, we furthermore show that we may always construct the sequence $\phi _N$ so that $E_\gamma (\phi _N) \rightarrow \gamma (x_0)$ as $N\rightarrow \infty$. This means that the Dirichlet energies of the solutions $u_N$ concentrate at point $x_0$ in the limit. These technical details are given in Lemma 5.7.
(vi) Since the previous step holds true at any point in *W*, one obtains by (i) a reconstruction method for γ in *W* (see Proposition 1.5). Furthermore, Lipschitz stability for exterior determination holds as a byproduct (see Proposition 1.4).

### Elliptic energy estimates for the fractional Schrödinger and conductivity equations

#### Definition 5.1

Let $s\in {\mathbb {R}}$. We say that a bilinear form $q:H^s({\mathbb {R}}^n)\times H^s({\mathbb {R}}^n)\rightarrow {\mathbb {R}}$ is local if q(u,v)=0 whenever *u*, *v* have disjoint supports.

One can see, for example, that if $P_1, P_2: H^s({\mathbb {R}}^n)\rightarrow L^2({\mathbb {R}}^n)$ are local bounded linear operators, that is ${{\,\textrm{supp}\,}}(P_ju) \subset {{\,\textrm{supp}\,}}(u)$, then $B(u,v):= \left\langle P_1u,P_2v \right\rangle$ is a local bilinear form. More generally, the related bilinear form would be local if the local linear operators $P_1$ and $P_2$ continuously map to some function space *X* and its dual $X^*$. This explains the used terminology, which was introduced earlier in [49]. In particular, the bilinear form related to $q_\gamma$ in Lemma 3.5 is local. The interest in the concept of local bilinear form is also motivated by foreseable future applications of the elliptic estimate introduced in Lemma 5.3.

#### Definition 5.2

Let $\Omega \subset {\mathbb {R}}^n$ be an open set such that $\Omega _e\ne \emptyset$ and $s\in {\mathbb {R}}_+$. For any local bounded bilinear form $q:H^s({\mathbb {R}}^n) \times H^s({\mathbb {R}}^n) \rightarrow {\mathbb {R}}$, we define the q-perturbed bilinear form $B_q:H^s({\mathbb {R}}^n)\times H^s({\mathbb {R}}^n)\rightarrow {\mathbb {R}}$ by

$$\begin{aligned} B_q(u,v):= \langle (-\Delta )^{s/2}u,(-\Delta )^{s/2}v\rangle +q(u,v) \end{aligned}$$

for all $u,v\in H^s({\mathbb {R}}^n)$. We say that $u\in H^s({\mathbb {R}}^n)$ is a weak solution to

$$\begin{aligned} \begin{aligned} ((-\Delta )^s+q)u&= F\quad \text {in}\quad \Omega ,\\ u&= f\quad \,\text {in}\quad \Omega _e \end{aligned} \end{aligned}$$

with $f\in H^s({\mathbb {R}}^n), F\in (\widetilde{H}^s(\Omega ))^*$, if $u-f\in \widetilde{H}^s(\Omega )$ and there holds

$$ B_q(u,\phi )=F(\phi ) $$

for all $$\phi \in \widetilde{H}^s(\Omega )$$.

#### Lemma 5.3

(Energy estimate for abstract Schrödinger equations) Let 0<s<1. Assume that $\Omega , W \subset {\mathbb {R}}^n$ are nonempty open sets such that |W|<∞ and $W\Subset \Omega _e$. Let $q:H^s({\mathbb {R}}^n) \times H^s({\mathbb {R}}^n) \rightarrow {\mathbb {R}}$ be a local bounded bilinear form and let $B_q$ be the q-perturbed bilinear form. Suppose that for any $F\in (\widetilde{H}^s(\Omega ))^*$ there is a unique solution $u_F\in H^s({\mathbb {R}}^n)$ of

13 $$\begin{aligned} \begin{aligned} ((-\Delta )^s+q)u&= F\quad \text {in}\quad \Omega ,\\ u&= 0\quad \,\,\text {in}\quad \Omega _e \end{aligned} \end{aligned}$$

and $u_F$ satisfies the estimate

14 $$\begin{aligned} \Vert u_F\Vert _{H^s({\mathbb {R}}^n)}\le C\Vert F\Vert _{(\widetilde{H}^s(\Omega ))^*} \end{aligned}$$

for some C>0 independent of *F*. Then for any $f\in C_c^{\infty }(W)$ there is a unique solution $u_f\in H^s({\mathbb {R}}^n)$ of

15 $$\begin{aligned} \begin{aligned} ((-\Delta )^s+q)u&= 0\quad \text {in}\quad \Omega ,\\ u&= f\quad \text {in}\quad \Omega _e \end{aligned} \end{aligned}$$

and there holds

$$\begin{aligned} \Vert u_f-f\Vert _{H^s({\mathbb {R}}^n)}\le C\Vert f\Vert _{L^2(W)} \end{aligned}$$

for some constant C>0 depending only on *n*, *s*, |*W*| and *dist*(W,Ω).

#### Proof

Let $$f\in C_c^{\infty }(W)$$ and $$F:=-B_q(f,\cdot )$$. Since $$B_q$$ is bounded, we know that $$F\in (\widetilde{H}^s(\Omega ))^*$$, and thus the unique solution $$u_F$$ of the problem (13) is well-defined. By construction it also holds that $$u_F+f$$ solves the problem (15). However, since the unique solution of (13) with vanishing inhomogeneity must necessarily be trivial, solutions to (15) are unique. Thus $$u_F + f $$ must be the *unique* solution $$u_f$$ to (15), and by (14)

$$ \Vert u_f-f\Vert _{H^s({\mathbb {R}}^n)}\le C\Vert B_q(f,\cdot )\Vert _{(\widetilde{H}^s(\Omega ))^*}. $$

Since *q* is local and $C_c^{\infty }(\Omega )$ is dense in $\widetilde{H}^s(\Omega )$, we have

$$\begin{aligned} \begin{aligned} \Vert u_f-f\Vert _{H^s({\mathbb {R}}^n)}&\le C\sup _{\begin{array}{c} \phi \in C_c^{\infty }(\Omega ):\\ \Vert \phi \Vert _{H^s({\mathbb {R}}^n)}=1 \end{array}}|\langle (-\Delta )^{s/2}f,(-\Delta )^{s/2}\phi \rangle |. \end{aligned} \end{aligned}$$

Moreover, by [36, Theorem 1.1, (g)]

$$\begin{aligned} |\langle (-\Delta )^{s/2}f,(-\Delta )^{s/2}\phi \rangle | = \frac{C_{n,s}}{2}\left| \int _{{\mathbb {R}}^{2n}}\frac{(f(x)-f(y))(\phi (x)-\phi (y))}{|x-y|^{n+2s}}dxdy\right| . \end{aligned}$$

If $(x,y)\in (\Omega ^c \times \Omega ^c)\cup (W^c\times W^c)$, the integrand function vanishes by the support conditions of *f* and ϕ. On the other hand $W \subset \Omega ^c$, and thus the integration set on the right hand side can be reduced to $(\Omega \times W) \cup (W\times \Omega )$. However, the integral over $\Omega \times W$ coincides to the one over $W\times \Omega$ by symmetry, and we are left with

$$\begin{aligned} |\langle (-\Delta )^{s/2}f,(-\Delta )^{s/2}\phi \rangle | = C_{n,s}\left| \int _{\Omega \times W}\frac{f(y)\phi (x)}{|x-y|^{n+2s}}\,dxdy\right| . \end{aligned}$$

By the Cauchy-Schwarz and Minkowski inequalities we have

$$ \begin{aligned}&\left| \int _{\Omega \times W}\frac{f(y)\phi (x)}{|x-y|^{n+2s}}\,dxdy\right| \\&\quad =\left| \int _{\Omega }\phi (x)\left( \int _{W}\frac{f(y)}{|x-y|^{n+2s}}\,dy\right) dx\right| \\&\quad \le \Vert \phi \Vert _{L^2(\Omega )}\left\| \int _{W}\frac{f(y)}{|x-y|^{n+2s}}\,dy\right\| _{L^2(\Omega )}\\&\quad \le \Vert \phi \Vert _{L^2(\Omega )}\left( \int _{W}\left( \int _{\Omega }\frac{|f(y)|^2}{|x-y|^{2n+4s}}\,dx\right) ^{1/2}\,dy\right) \\&\quad =\Vert \phi \Vert _{L^2(\Omega )}\left( \int _{W}|f(y)|\left( \int _{\Omega }\frac{1}{|x-y|^{2n+4s}}\,dx\right) ^{1/2}\,dy\right) . \end{aligned} $$

Observe that since $W\Subset \Omega _e$, for $(x,y)\in \Omega \times W$ it holds $|x-y|\ge \text{ dist }(\Omega ,W)=:r>0$, and thus

$$ \begin{aligned} \int _{\Omega }\frac{1}{|x-y|^{2n+4s}}\,dx&\le \int _{B_r(y)^c}\frac{1}{|x-y|^{2n+4s}}\,dx=\frac{\omega _n}{(n+4s)r^{n+4s}}. \end{aligned} $$

Hence, by Hölder’s inequality we get

$$ \begin{aligned} \left| \int _{\Omega \times W}\frac{f(y)\phi (x)}{|x-y|^{n+2s}}\,dxdy\right|&\le \sqrt{\frac{\omega _n}{(n+4s)r^{n+4s}}}|W|^{1/2}\Vert f\Vert _{L^2(W)}\Vert \phi \Vert _{L^2(\Omega )}, \end{aligned} $$

and eventually

$$ \Vert u_f-f\Vert _{H^s({\mathbb {R}}^n)}\le C C_{n,s} \sqrt{\frac{\omega _n}{(n+4s)r^{n+4s}}}|W|^{1/2}\Vert f\Vert _{L^2(W)} $$

where C>0 is the constant from the elliptic a priori estimate (14). □

#### Corollary 5.4

(Energy estimate for conductivity equations) Let 0<s<min(1,n/2). Suppose that $\Omega \subset {\mathbb {R}}^n$ is an open set which is bounded in one direction, $W\subset \Omega _e$ an open set with finite measure and positive distance from Ω. Assume that $\gamma \in L^{\infty }({\mathbb {R}}^n)$ with conductivity matrix Θ and background deviation *m* satisfies $\gamma (x)\ge \gamma _0>0$ and $m\in H^{2s,\frac{n}{2s}}({\mathbb {R}}^n)$. Then for any $f\in C_c^{\infty }(W)$ the associated unique solution $u_f\in H^s({\mathbb {R}}^n)$ of

$$ \begin{aligned} {{\,\textrm{div}\,}}_s(\Theta \nabla ^s u)&= 0\quad \text {in}\quad \Omega ,\\ u&= f\quad \text {in}\quad \Omega _e, \end{aligned} $$

satisfies the estimate

$$ \Vert u_f-f\Vert _{H^s({\mathbb {R}}^n)}\le C\Vert f\Vert _{L^2(W)} $$

for some C>0 depending only on $n,s,\gamma _0,\Vert \gamma \Vert _{L^{\infty }({\mathbb {R}}^n)},|W|$, the distance between *W* and Ω, and the Poincaré constant of Ω.

#### Proof

First note that by [49, Lemma 8.10] for any $f\in C_c^{\infty }(W)$ there is a unique solution $u_f\in H^s({\mathbb {R}}^n)$ solving

$$ \begin{aligned} {{\,\textrm{div}\,}}_s(\Theta \nabla ^s u)&= 0\quad \text {in}\quad \Omega ,\\ u&= f\quad \text {in}\quad \Omega _e \end{aligned} $$

and since they are constructed via the Lax–Milgram theorem (cf. [49, Lemma 8.10]) there holds

$$ \begin{aligned} \Vert u_f-f\Vert _{H^s({\mathbb {R}}^n)}&\le C\Vert B_{\gamma }(f,\cdot )\Vert _{(\widetilde{H}^s(\Omega ))^*} \end{aligned} $$

where C(s,Ω)>0 depends on the fractional Poincaré constant of Ω.

By [49, Remark 8.8] and density of $C_c^{\infty }(\Omega )$ in $\widetilde{H}^s(\Omega )$ this gives

$$\small { \begin{aligned}&\Vert u_f-f\Vert _{H^s({\mathbb {R}}^n)}\le C\sup _{\begin{array}{c} \phi \in C_c^{\infty }(\Omega ):\\ \Vert \phi \Vert _{H^s({\mathbb {R}}^n)}=1 \end{array}}|\langle \Theta \nabla ^sf,\nabla ^s\phi \rangle _{L^2({\mathbb {R}}^{2n})}|\\&=C\sup _{\begin{array}{c} \phi \in C_c^{\infty }(\Omega ):\\ \Vert \phi \Vert _{H^s({\mathbb {R}}^n)}=1 \end{array}}|\langle (-\Delta )^{s/2}(\gamma ^{1/2}f),(-\Delta )^{s/2}(\gamma ^{1/2}\phi )\rangle _{L^2({\mathbb {R}}^{n})}+\langle q\gamma ^{1/2}f,\gamma ^{1/2}\phi \rangle _{L^2({\mathbb {R}}^n)}|\\&=C\sup _{\begin{array}{c} \phi \in C_c^{\infty }(\Omega ):\\ \Vert \phi \Vert _{H^s({\mathbb {R}}^n)}=1 \end{array}}|\langle (-\Delta )^{s/2}(\gamma ^{1/2}f),(-\Delta )^{s/2}(\gamma ^{1/2}\phi )\rangle _{L^2({\mathbb {R}}^{n})}|, \end{aligned}} $$

where $q:= -\frac{(-\Delta )^sm}{\gamma ^{1/2}}\in L^{n/2s}({\mathbb {R}}^n)$, and the last equality follows from the fact that *f* and ϕ have disjoint support.

Let $(\rho _{\epsilon })_{\epsilon >0}\subset C_c^{\infty }({\mathbb {R}}^n)$ be the standard mollifiers and define the sequences $\gamma _{\epsilon }^{1/2}:=\gamma ^{1/2}*\rho _{\epsilon }\in C_b^{\infty }({\mathbb {R}}^n)$ and $m_{\epsilon }:=m*\rho _{\epsilon }\in C_b^{\infty }({\mathbb {R}}^n)$. Observe that $m_{\epsilon }=\gamma _{\epsilon }^{1/2}-1$, since $\int _{{\mathbb {R}}^n}\rho _{\epsilon }\,dx=1$. By the properties of mollification in $L^p$ spaces and the Bessel potential operator, we deduce

(I) $$\gamma _{\epsilon }^{1/2}\overset{*}{\rightharpoonup }\ \gamma ^{1/2}$$ in $$L^{\infty }({\mathbb {R}}^n)$$,
(II) $$0<\gamma _0^{1/2}\le \gamma _{\epsilon }^{1/2}\in L^{\infty }({\mathbb {R}}^n)$$ with $$\Vert \gamma _{\epsilon }^{1/2}\Vert _{L^{\infty }({\mathbb {R}}^n)}\le \Vert \gamma \Vert _{L^{\infty }({\mathbb {R}}^n)}^{1/2}$$,
(III) $$m_{\epsilon }\rightarrow m$$ in $$H^{2s,\frac{n}{2s}}({\mathbb {R}}^n)$$,
(IV) $$m_{\epsilon }\in L^{\infty }({\mathbb {R}}^n)$$ with $$\Vert m_{\epsilon }\Vert _{L^{\infty }({\mathbb {R}}^n)}\le \Vert m\Vert _{L^{\infty }({\mathbb {R}}^n)}\le 1+\Vert \gamma \Vert _{L^{\infty }({\mathbb {R}}^n)}^{1/2}$$.

Using [49, Corollary A.7], we deduce

$$ m_{\epsilon }u\rightarrow mu\quad \text {in}\quad H^s({\mathbb {R}}^n) $$

as $\epsilon \rightarrow 0$ for any $u\in H^s({\mathbb {R}}^n)$ and therefore by continuity of the fractional Laplacian we have

$$ \begin{aligned}&\langle (-\Delta )^{s/2}(\gamma _{\epsilon }^{1/2}f),(-\Delta )^{s/2}(\gamma _{\epsilon }^{1/2}\phi )\rangle _{L^2({\mathbb {R}}^n)}\\&\quad =\langle (-\Delta )^{s/2}((m_{\epsilon }+1)f),(-\Delta )^{s/2}((m_{\epsilon }+1)\phi )\rangle _{L^2({\mathbb {R}}^n)}\\&\quad \rightarrow \langle (-\Delta )^{s/2}(\gamma ^{1/2}f),(-\Delta )^{s/2}(\gamma ^{1/2}\phi )\rangle _{L^2({\mathbb {R}}^n)} \end{aligned} $$

as $\epsilon \rightarrow 0$. Now on the $L^2$ inner product on the left hand side we can apply the calculation of Lemma 5.3 and deduce by using (II) the estimate

$$\begin{aligned}&|\langle (-\Delta )^{s/2}(\gamma _{\epsilon }^{1/2}f),(-\Delta )^{s/2}(\gamma _{\epsilon }^{1/2}\phi )\rangle _{L^2({\mathbb {R}}^n)}|\\&\quad \le CC_{n,s}\Vert \gamma \Vert _{L^{\infty }({\mathbb {R}}^n)}\sqrt{\frac{\omega _n}{(n+4s)r^{n+4s}}}|W|^{1/2}\Vert f\Vert _{L^2(W)}\Vert \phi \Vert _{L^2(\Omega )}. \end{aligned} $$

This shows

$$ \Vert u_f-f\Vert _{H^s({\mathbb {R}}^n)}\le CC_{n,s}\Vert \gamma \Vert _{L^{\infty }({\mathbb {R}}^n)}\sqrt{\frac{\omega _n}{(n+4s)r^{n+4s}}}|W|^{1/2}\Vert f\Vert _{L^2(W)}. $$

□

### Construction of exterior conditions with an energy concetration property

For any 0<s<min(1,n/2) and positive conductivity $\gamma \in L^{\infty }({\mathbb {R}}^n)$ with conductivity matrix Θ we denote the associated energy by

$$ E_{\gamma }(u):= B_{\gamma }(u,u)= \int _{{\mathbb {R}}^{2n}}\Theta \nabla ^su\cdot \nabla ^su\,dxdy $$

for all $$u\in H^s({\mathbb {R}}^n)$$.

#### Lemma 5.5

(Exterior conditions) Let $\Omega \subset {\mathbb {R}}^n$ be open with nonempty exterior, 0<s<min(1,n/2), $x_0\in W \subset \Omega _e$ for an open set *W*, and $M\in {\mathbb {N}}$. There exists a sequence $(\phi _N)_{N\in {\mathbb {N}}}\subset C_c^{\infty }(W)$ such that

(i) for all $N\in {\mathbb {N}}$ it holds $\Vert \phi _N\Vert _{H^s({\mathbb {R}}^n)}=1$,
(ii) for all t≥-M there exist constants $C_{t,s},C'_{t,s}>0$ such that
  $$C'_{t,s}N^t\le \Vert \phi _N\Vert _{H^{t+s}({\mathbb {R}}^n)}\le C_{t,s}N^{t} \quad \text{ for } \text{ all } N\in {\mathbb {N}}, $$
(iii) $${{\,\textrm{supp}\,}}(\phi _N)\rightarrow \{x_0\}$$ as $$N\rightarrow \infty $$.

Kohn and Vogelius proved a similar result in their celebrated work on boundary determination for the conductivity equation (cf. [34, Lemma 1]) for the Sobolev spaces $H^s(\partial \Omega )$, where $\Omega \subset {\mathbb {R}}^n$ is an open bounded set with smooth boundary. For the sake of completeness, we provide a proof of this related result in Appendix A.

#### Remark 5.6

One may rescale the functions given by Lemma 5.5 so that the properties (i) and (ii) hold true in the equivalent norms of $W^s({\mathbb {R}}^n)$, defined in (7). In the following proofs, we will use sequences $(\phi _N)_{N\in {\mathbb {N}}}\subset C_c^{\infty }(W)$ such that $\Vert \phi _N \Vert _{L^2({\mathbb {R}}^n)} \rightarrow 0$ as $N \rightarrow \infty$ and $\Vert \phi _N \Vert _{W^s({\mathbb {R}}^n)}=1$.

#### Lemma 5.7

(Energy concentration property) Let $\Omega \subset {\mathbb {R}}^n$ be an open set which is bounded in one direction and 0<s<min(1,n/2). Assume that $\gamma \in L^{\infty }({\mathbb {R}}^n)$ with background deviation $m_{\gamma }$ satisfy $\gamma (x)\ge \gamma _0>0$ and $m_{\gamma }\in H^{2s,\frac{n}{2s}}({\mathbb {R}}^n)$. If $W \subset \Omega _e$ is a nonempty, bounded, open set such that $\gamma \in C(W)$, $x_0\in W$, then for any sequence $(\phi _N)_{N \in {\mathbb {N}}}$, which satisfies the properties of Lemma 5.5 there holds

$$ \gamma (x_0)=\lim _{N\rightarrow \infty }E_{\gamma }(\phi _N). $$

#### Proof

Let $\phi _N$ be such that they have the properties given in Remark 5.6 of Lemma 5.5. By the product rule for the fractional gradient (8),

16 $$\begin{aligned} |\nabla ^s(\phi _N\psi )|^2\le 2\left( |\phi _N(x)|^2|\nabla ^s\psi (x,y)|^2+|\psi (y)|^2|\nabla ^s\phi _N(x,y)|^2\right) . \end{aligned}$$

If $\psi \in C^1_b({\mathbb {R}}^n)$, then by the mean value theorem

$$ \begin{aligned}&\int _{{\mathbb {R}}^n}|\nabla ^s\psi (x,y)|^2\,dy \\&\quad \lesssim \int _{B_1(x)}\frac{|\psi (x)-\psi (y)|^2}{|x-y|^{n+2s}}\,dy+\int _{{\mathbb {R}}^n \setminus B_1(x)}\frac{|\psi (x)-\psi (y)|^2}{|x-y|^{n+2s}}\,dy\\&\quad \lesssim \Vert \nabla \psi \Vert ^2_{L^{\infty }({\mathbb {R}}^n)}\int _{B_1(x)}\frac{dy}{|x-y|^{n+2s-2}}+\Vert \psi \Vert ^2_{L^{\infty }({\mathbb {R}}^n)}\int _{{\mathbb {R}}^n \setminus B_1(x)}\frac{dy}{|x-y|^{n+2s}}\\&\quad \lesssim \left( \int _{B_1(0)}\frac{dy}{|y|^{n-2(1-s)}}+\int _{{\mathbb {R}}^n\setminus B_1(0)}\frac{dy}{|y|^{n+2s}}\right) \Vert \psi \Vert _{C^1({\mathbb {R}}^n)}^2\\&\quad \lesssim \Vert \psi \Vert _{C^1({\mathbb {R}}^n)}^2 \end{aligned} $$

for all $x\in {\mathbb {R}}^n$. By (ii) of Lemma 5.5 with t=-s we have $\Vert \phi _N\Vert _{L^2({\mathbb {R}}^n)}\rightarrow 0$ as $N\rightarrow \infty$, and thus

17 $$\begin{aligned} \int _{{\mathbb {R}}^{2n}}|\phi _N(x)|^2|\nabla ^s\psi (x,y)|^2\,dxdy\lesssim \Vert \psi \Vert _{C^1({\mathbb {R}}^n)}^2\Vert \phi _N\Vert _{L^2({\mathbb {R}}^n)}^2\rightarrow 0 \end{aligned}$$

for all $\psi \in C_c^1({\mathbb {R}}^n)$ as $N\rightarrow \infty$. Consider now a sequence of functions $(\eta _M)_{M\in {\mathbb {N}}}\subset C^1_c({\mathbb {R}}^n)$ such that for all $M\in {\mathbb {N}}$ it holds $0\le \eta _M\le 1$, $\eta _M|_{Q_{1/2M}(x_0)}=1$ and $\eta _M|_{(Q_{1/M}(x_0))^c}=0$, where the cube $Q_r(x)$ is defined for all $x\in {\mathbb {R}}^n$, r>0 as

$$ Q_r(x):=\{y\in {\mathbb {R}}^n;\,|y_i-x_i|<r\quad \text {for all}\quad i=1,\ldots ,n\}. $$

Then for all $M\in {\mathbb {N}}$

18 $$\begin{aligned} \begin{aligned}&\limsup _{N\rightarrow \infty }\left| \int _{{\mathbb {R}}^{2n}}(\gamma ^{1/2}(y)-\gamma ^{1/2}(x_0))\gamma ^{1/2}(x)|\nabla ^s\phi _N|^2\,dxdy\right| \\&\quad \le \Vert \gamma \Vert _{L^{\infty }({\mathbb {R}}^n)}^{1/2}\limsup _{N\rightarrow \infty }\int _{{\mathbb {R}}^{2n}}|\gamma ^{1/2}(y)-\gamma ^{1/2}(x_0)|\,|\nabla ^s\phi _N(x,y)|^2\,dxdy\\&\quad =\Vert \gamma \Vert _{L^{\infty }({\mathbb {R}}^n)}^{1/2}\limsup _{N\rightarrow \infty }\int _{{\mathbb {R}}^{2n}}|\gamma ^{1/2}(y)-\gamma ^{1/2}(x_0)|\,|\nabla ^s(\phi _N\eta _M)(x,y)|^2\,dxdy\\&\quad \lesssim \Vert \gamma \Vert _{L^{\infty }({\mathbb {R}}^n)}^{1/2}\limsup _{N\rightarrow \infty }\int _{{\mathbb {R}}^{2n}}|\gamma ^{1/2}(y)-\gamma ^{1/2}(x_0)|\,|\eta _M(y)|^2\,|\nabla ^s\phi _N(x,y)|^2\,dxdy\\&\quad \le \Vert \gamma \Vert _{L^{\infty }({\mathbb {R}}^n)}^{1/2}\Vert \gamma ^{1/2}-\gamma ^{1/2}(x_0)\Vert _{L^{\infty }(Q_{1/M}(x_0))} \end{aligned} \end{aligned}$$

where we used that $\phi _N\eta _M=\phi _N$ for sufficiently large *N*, the inequality (16), the subadditivity of limsup, the vanishing of the limit from the formula (17) and the property $\Vert \phi _N\Vert _{W^s({\mathbb {R}}^n)}=1$ in the last estimate. By taking the limit $M \rightarrow \infty$ and using that γ is continuous, we obtain that

19 $$\begin{aligned} \lim _{N\rightarrow \infty } \int _{{\mathbb {R}}^{2n}}(\gamma ^{1/2}(y)-\gamma ^{1/2}(x_0))\gamma ^{1/2}(x)|\nabla ^s\phi _N|^2\,dxdy = 0. \end{aligned}$$

We may now calculate that

$$\begin{aligned} \begin{aligned} \gamma (x_0) =&\lim _{N\rightarrow \infty }\int _{{\mathbb {R}}^{2n}}\gamma ^{1/2}(x)(\gamma ^{1/2}(y)-\gamma ^{1/2}(x_0))|\nabla ^s\phi _N|^2\,dxdy\\&+\gamma ^{1/2}(x_0)\lim _{N\rightarrow \infty }\int _{{\mathbb {R}}^{2n}}(\gamma ^{1/2}(x)-\gamma ^{1/2}(x_0))|\nabla ^s\phi _N|^2\,dxdy\\&+\gamma (x_0)\lim _{N\rightarrow \infty }\int _{{\mathbb {R}}^{2n}}|\nabla ^s\phi _N|^2\,dxdy\\ =&\lim _{N\rightarrow \infty }\int _{{\mathbb {R}}^{2n}}\gamma ^{1/2}(x)(\gamma ^{1/2}(y)-\gamma ^{1/2}(x_0))|\nabla ^s\phi _N|^2\,dxdy\\&+\gamma ^{1/2}(x_0)\lim _{N\rightarrow \infty }\int _{{\mathbb {R}}^{2n}}\gamma ^{1/2}(x)|\nabla ^s\phi _N|^2\,dxdy\\ =&\lim _{N\rightarrow \infty }\int _{{\mathbb {R}}^{2n}}\gamma ^{1/2}(x)\gamma ^{1/2}(y)|\nabla ^s\phi _N|^2\,dxdy\\ =&\lim _{N\rightarrow \infty }\langle \Theta _{\gamma }\nabla ^s\phi _N,\nabla ^s\phi _N\rangle _{L^2({\mathbb {R}}^{2n})}\\ =&\lim _{N\rightarrow \infty } E_\gamma (\phi _N) \end{aligned} \end{aligned}$$

where the first limit on the right hand side must vanish by (19), the second limit must vanish by the argument given in (18), and $\lim _{N\rightarrow \infty }\int _{{\mathbb {R}}^{2n}}|\nabla ^s\phi _N|^2=1$ holds as $\Vert (-\Delta )^{s/2}\phi _N \Vert _{L^2({\mathbb {R}}^n)}^2+\Vert \phi _N \Vert _{L^2({\mathbb {R}}^n)}^2 =1$ for every $N \in {\mathbb {N}}$ and $\Vert \phi _N \Vert _{L^2({\mathbb {R}}^n)} \rightarrow 0$ as $N\rightarrow \infty$. □

#### Remark 5.8

The proof of Lemma 5.7 shows that it is sufficient if the sequence $\phi _N$ only satisfies a weaker condition $\Vert \phi _N \Vert _{L^2({\mathbb {R}}^n)} \rightarrow 0$ as $N \rightarrow \infty$ instead of Lemma 5.5 (ii). This observation also carries over to the exterior determination results given in Section 5.3 due to the $L^2$ energy estimates of Lemma 5.3 (cf. the proof of Proposition 1.5).

### Proofs of the exterior determination results

Given the preparations made in Sections 5.1 and 5.2, we may now prove our results on exterior determination for the fractional conductivity equation.

#### Proof of Proposition 1.5

Let $\phi _N \in C_c^\infty (W)$ be a sequence of functions as constructed in Remark 5.6 of Lemma 5.5. Let $u_N\in H^s({\mathbb {R}}^n)$ be the unique solution to the fractional conductivity equation (9) with exterior value $\phi _N$. We show that one can approximate the energies $E_\gamma (u_N)$ by the energies of the exterior conditions $\phi _N$.

First assume $u_f\in H^s({\mathbb {R}}^n)$ is a solution to the fractional conductivity equation with exterior value $f \in C_c^\infty (W)$. Then we have the following energy decomposition

20 $$\begin{aligned} \begin{aligned} E_\gamma (u_f)&= \langle \Theta _{\gamma } \nabla ^su_f,\nabla ^su_f\rangle _{L^2({\mathbb {R}}^{2n})}\\&= \langle \Theta _{\gamma }\nabla ^s((u_f-f)+f),\nabla ^s((u_f-f)+f)\rangle _{L^2({\mathbb {R}}^{2n})} \\&= E_\gamma (u_f-f) + 2\langle \Theta _\gamma \nabla ^sf,\nabla ^s(u_f-f)\rangle _{L^2({\mathbb {R}}^{2n})}+ E_\gamma (f). \end{aligned} \end{aligned}$$

By (ii) of Lemma 5.5, we know that $\phi _N\rightarrow 0$ in $L^2({\mathbb {R}}^n)$ and, therefore, by Corollary 5.4 it follows

$$\begin{aligned} \Vert u_{N}-\phi _N \Vert _{H^s({\mathbb {R}}^n)} \rightarrow 0 \end{aligned}$$

as $N\rightarrow \infty$. Using the decomposition (20), the boundedness of the bilinear form, the Cauchy-Schwarz inequality and the boundedness of the fractional gradient, we see that the first two terms in (20) vanish as $N\rightarrow \infty$. Thus by Lemma 5.7

$$\begin{aligned} \lim _{N\rightarrow \infty } E_{\gamma }(u_{N}) = \lim _{N\rightarrow \infty } E_\gamma (\phi _N) = \gamma (x_0). \end{aligned}$$

□

Proposition 1.5 readily implies Theorem 1.2.

#### Proof of Theorem 1.2

Fix $x_0 \in W$. Let $\phi _N$ be as in Proposition 1.5, and let $u_{N,i}$ be the solution to the conductivity equation (9) with conductivity $\gamma _i$, i=1,2, and exterior condition $\phi _N$. We have by the definitions of the DN maps and Dirichlet energies that

$$ E_{\gamma _i}(u_{N,i})=B_{\gamma _i}(u_{N,i},u_{N,i})=B_{\gamma _i}(u_{N,i},\phi _N)=\langle \Lambda _{\gamma _i} \phi _N,\phi _N\rangle _{X^*\times X}. $$

Now Proposition 1.5, $\phi _N \in C_c^\infty (W)$ and the fact that $\Lambda _{\gamma _1}\phi _N|_W = \Lambda _{\gamma _2}\phi _N|_W$ for every $N \in {\mathbb {N}}$ let us conclude that

$$\begin{aligned} \gamma _1(x_0) = \lim _{N\rightarrow \infty }E_{\gamma _1}(u_{N,1}) = \lim _{N\rightarrow \infty }E_{\gamma _2}(u_{N,2}) = \gamma _2(x_0). \end{aligned}$$

□

We conclude with a stability estimate for the exterior determination.

#### Proof of Proposition 1.4

Proceeding as in the proof of Theorem 1.2, we can write

$$ \gamma _i(x_0)=\lim _{N\rightarrow \infty }\langle \Lambda _{\gamma _i}\phi _N,\phi _N\rangle _{X^*\times X} $$

for i=1,2. This gives

$$ \begin{aligned} |\gamma _1(x_0)-\gamma _2(x_0)|&=\lim _{N\rightarrow \infty }|\langle \Lambda _{\gamma _1}\phi _N,\phi _N\rangle _{X^*\times X}-\langle \Lambda _{\gamma _2}\phi _N,\phi _N\rangle _{X^*\times X}|\\&=\lim _{N\rightarrow \infty }|\langle (\Lambda _{\gamma _1}-\Lambda _{\gamma _2})\phi _N,\phi _N\rangle _{X^*\times X}|\\&\le \lim _{N\rightarrow \infty }\Vert (\Lambda _{\gamma _1}-\Lambda _{\gamma _2})\phi _N\Vert _{X^*}\Vert \phi _N\Vert _X\\&\le \Vert \Lambda _{\gamma _1}-\Lambda _{\gamma _2}\Vert _{X\rightarrow X^*}\lim _{N\rightarrow \infty }\Vert \phi _N\Vert _{X}^2\\&\le \Vert \Lambda _{\gamma _1}-\Lambda _{\gamma _2}\Vert _{X\rightarrow X^*}\lim _{N\rightarrow \infty }\Vert \phi _N\Vert _{H^s({\mathbb {R}}^n)}^2 \\&\le 2^s \Vert \Lambda _{\gamma _1}-\Lambda _{\gamma _2}\Vert _{X\rightarrow X^*} \lim _{N\rightarrow \infty }\Vert \phi _N\Vert _{W^s({\mathbb {R}}^n)}^2 \end{aligned} $$

Since this estimate holds uniformly for all $x_0\in W$ and $\Vert \phi _N\Vert _{W^s({\mathbb {R}}^n)}^2=1$ for all $N \in {\mathbb {N}}$, we have shown

$$ \Vert \gamma _1-\gamma _2\Vert _{L^{\infty }(W)}\le 2^s\Vert \Lambda _{\gamma _1}-\Lambda _{\gamma _2}\Vert _{X\rightarrow X^*}. $$

□

#### Remark 5.9

If we define the norm of *X* with respect to the norm $W^s({\mathbb {R}}^n)$, then the stability estimate of Proposition 1.4 holds with the constant 1 in these modified norms.

## Data Availability

Data sharing not applicable to this article as no datasets were generated or analysed during the current study.

## Appendix A. Proof of Lemma 5.5

### Proof

First assume $x_0=0$. Let $\psi \in C_c^{\infty }({\mathbb {R}})$ satisfy ${{\,\textrm{supp}\,}}(\psi )\subset [-1,1]$, $\psi \ne 0$ and

21 $$\begin{aligned} \int _{-1}^1r^k\psi (r)\,dr=0\quad \text {for all}\quad 0\le k\le M-1. \end{aligned}$$

For any $N\in {\mathbb {N}}$ define

$$ \psi _N(x):= \prod _{i=1}^n\psi (Nx_i)\in C_c^{\infty }({\mathbb {R}}^n). $$

By construction ${{\,\textrm{supp}\,}}(\psi _N)\subset [-1/N,1/N]^n$, and thus ${{\,\textrm{supp}\,}}(\psi _N)\rightarrow \{0\}$ as $N\rightarrow \infty$. For all $k\in {\mathbb {N}}_0$ we have

$$\begin{aligned} \begin{aligned} \Vert \psi _N\Vert ^2_{H^k({\mathbb {R}}^n)}&=\sum _{|\alpha |\le k}\Vert \partial ^{\alpha }\psi _N\Vert ^2_{L^2({\mathbb {R}}^n)} =\sum _{|\alpha |\le k}N^{2|\alpha |}\prod _{i=1}^n\Vert (\partial ^{\alpha _i}\psi )(N\cdot )\Vert ^2_{L^2({\mathbb {R}})} \\  &=\sum _{|\alpha |\le k}N^{2|\alpha |-n}\prod _{i=1}^n\Vert \partial ^{\alpha _i}\psi \Vert ^2_{L^2({\mathbb {R}})}, \end{aligned} \end{aligned}$$

and thus using the fact that $\Vert \partial ^{\alpha _i}\psi \Vert _{L^2({\mathbb {R}})} \le \Vert \psi \Vert _{H^k({\mathbb {R}})}$ for all i=1,...,n we get the upper bound

$$\begin{aligned} \Vert \psi _N\Vert _{H^k({\mathbb {R}}^n)}\le C_kN^{k-n/2}\Vert \psi \Vert _{H^k({\mathbb {R}})}^{n} \le C_kN^{k-n/2}. \end{aligned}$$

A lower bound can be obtained by estimating the sum from below by the term corresponding to the multi-index α=(0,...,0,k):

$$ \Vert \psi _N\Vert ^2_{H^k({\mathbb {R}}^n)}\ge N^{2k-n}\Vert \psi \Vert ^{2(n-1)}_{L^2({\mathbb {R}})}\Vert \partial ^k\psi \Vert _{L^2({\mathbb {R}})}^2>0, $$

where the strict positivity follows from the Poincaré inequality and $\psi \ne 0$. This shows that for all $k\in {\mathbb {N}}_0$ there are constants $C_k, C'_k>0$ verifying the estimate

22 $$\begin{aligned} C'_kN^{k-n/2}\le \Vert \psi _N\Vert _{H^k({\mathbb {R}}^n)}\le C_kN^{k-n/2} \end{aligned}$$

Next, we wish to extend the estimate (22) from $k\in {\mathbb {N}}_0$ to some negative integers. Since

$$ \begin{aligned} \Vert \psi _N\Vert _{H^{-k}({\mathbb {R}}^n)}&=\sup _{v\in H^k({\mathbb {R}}^n): \,\Vert v\Vert _{H^k({\mathbb {R}}^n)}=1}|\langle \psi _N,v\rangle |\\&\ge |\langle \psi _N,\psi _N/\Vert \psi _N\Vert _{H^k({\mathbb {R}}^n)}\rangle |=\frac{\Vert \psi _N\Vert _{L^2({\mathbb {R}}^n)}^2}{\Vert \psi _N\Vert _{H^k({\mathbb {R}}^n)}}, \end{aligned} $$

applying the formula (22) twice we immediately obtain the lower bound

$$\begin{aligned} \Vert \psi _N\Vert _{H^{-k}({\mathbb {R}}^n)} \ge C'_{-k}N^{-k-n/2}. \end{aligned}$$

We now produce an upper bound. Since ψ is smooth and compactly supported, by the Paley–Wiener theorem $\hat{\psi }$ has an entire extension, and thus we can write

$$\begin{aligned} \hat{\psi }(z)= \sum _{j=0}^\infty \frac{z^j}{j!}\partial ^j_{\xi }\hat{\psi }(0) \end{aligned}$$

for all $z \in {\mathbb {C}}$. However, (21) implies that $\partial ^j_{\xi }\hat{\psi }(0)=0$ for all j∈[0,M-1], and therefore

$$\begin{aligned} \hat{\psi }(z) = z^M \sum _{j=0}^\infty \frac{z^j}{(j+M)!}\partial ^{j+M}_{\xi }\hat{\psi }(0). \end{aligned}$$

One sees immediately that the function of *z* defined by the series

$$\begin{aligned} \hat{\phi }(z)= \sum _{j=0}^\infty \frac{z^j}{(j+M)!}\partial ^{j+M}_{\xi }\hat{\psi }(0) \end{aligned}$$

is a meromorphic function in ${\mathbb {C}}\setminus \{0\}$ with a pole at the origin. Since this pole is removable, $\hat{\phi }$ extends into an entire function satisfying the assumptions of the Paley–Wiener theorem whose restriction to the real line (i.e. the Fourier transform of ϕ) decays faster than any polynomial. Therefore, there exists $\phi \in C_c^\infty ({\mathbb {R}}^n)$ with ${{\,\textrm{supp}\,}}(\phi ) \subset [-1,1]$ such that $\psi = (-i)^M\partial ^M_r\phi$. In the following computations, we simply redefine ϕ so that the constant term $(-i)^M$ is included into it.

Consider now the multi-index α=(k,0,...,0) of length k∈(0,M]. One can write

$$\begin{aligned} \psi _N(x)&= \prod _{i=1}^n \psi (Nx_i) = \psi (Nx_1)\prod _{i=2}^n\psi (Nx_i) \\  &= N^{-k}\partial _{x_1}^k((\partial ^{M-k}\phi )(Nx_1))\prod _{i=2}^n\psi (Nx_i) \\  &= N^{-k}D^\alpha _x\left( (\partial ^{M-k}\phi )(Nx_1)\prod _{i=2}^n\psi (Nx_i) \right) , \end{aligned}$$

which now ensures

$$\begin{aligned} \Vert \psi _N\Vert _{H^{-k}(\mathbb {R}^n)}&\le N^{-k}\Vert (\partial ^{M-k}\phi )(Nx_1)\prod _{i=2}^n\psi (Nx_i) \Vert _{L^2(\mathbb {R}^n)} \\  &= N^{-k}\Vert (\partial ^{M-k}\phi )(N\cdot )\Vert _{L^2({\mathbb {R}})}\Vert \psi (N\cdot )\Vert ^{n-1}_{L^2({\mathbb {R}})}\\  &= N^{-k-n/2} \Vert \partial ^{M-k}\phi \Vert _{L^2({\mathbb {R}})}\Vert \psi \Vert ^{n-1}_{L^2({\mathbb {R}})} \end{aligned}$$

and eventually $\Vert \psi _N\Vert _{H^{-k}({\mathbb {R}}^n)}\le C_{-k}N^{-k-n/2}$ for some $C_{-k}>0$ and 0<k≤M. Here we have used the characterization of the negative order Sobolev space $H^{-k}(\mathbb {R}^n)$ as the space of distributions which can be written in the form $f=\sum _{|\alpha |\le k} D^\alpha f_\alpha$ with $f_\alpha \in L^2(\mathbb {R}^n)$, equipped with the norm $\Vert f\Vert _{H^{-k}({\mathbb {R}}^n)} = \inf \{ \sum _{|\alpha |\le k} \Vert f_\alpha \Vert _{L^2({\mathbb {R}}^n)}\}$, the infimum being taken over all such possible expressions of *f*. Hence

23 $$\begin{aligned} C'_{-k}N^{-k-n/2}\le \Vert \psi _N\Vert _{H^{-k}({\mathbb {R}}^n)}\le C_{-k} N^{-k-n/2} \end{aligned}$$

for some constants $C_{-k},C'_{-k}>0$ and 0<k≤M.

Next, we seek to the extend estimates (22) and (23) to all sufficiently large real numbers. Let $k\in {\mathbb {Z}}$ be such that t+s∈(k,k+1). Then there exists $\theta \in (0,1)$ such that $t+s=\theta k+(1-\theta )(k+1)$. Using interpolation in Bessel potential spaces (cf. [6, Theorem 6.4.5]), (22) and (23), we deduce

24 $$\begin{aligned} \Vert \psi _N\Vert _{H^{t+s}({\mathbb {R}}^n)}\le C_{t,s}\left( N^{k-n/2}\right) ^{\theta }\left( N^{k+1-n/2}\right) ^{1-\theta }=C_{t,s}N^{t+s-n/2} \end{aligned}$$

for all $t\in {\mathbb {R}}$ such that t+s≥-M, which gives the wanted upper bound. For the lower bound, we shall divide the cases t+s≥0 and t+s<0.

Assume first t+s≥0. Using $\Vert u\Vert _{H^{t+s}({\mathbb {R}}^n)}\sim \Vert u\Vert _{L^2({\mathbb {R}}^n)}+\Vert (-\Delta )^{\frac{t+s}{2}}u\Vert _{L^2({\mathbb {R}}^n)}$ and $\widehat{\psi _N}(\xi )=N^{-n}\widehat{\psi _1}(\xi /N)$ we obtain

25 $$\begin{aligned} \begin{aligned} \Vert \psi _N\Vert _{H^{t+s}({\mathbb {R}}^n)}&\ge C_{t,s}\Vert (-\Delta )^{\frac{t+s}{2}}\psi _N\Vert _{L^2(\mathbb {R}^n)}=C_{t,s}\Vert |\xi |^{t+s}\widehat{\psi _N}\Vert _{L^2({\mathbb {R}}^n)}\\&=C_{t,s}N^{-n}\Vert |\xi |^{t+s}\widehat{\psi _1}(\cdot /N)\Vert _{L^2({\mathbb {R}}^n)}\\&=C_{t,s}N^{t+s-n/2}\Vert (-\Delta )^{\frac{t+s}{2}}\psi _1\Vert _{L^2({\mathbb {R}}^n)}. \end{aligned} \end{aligned}$$

If instead t+s<0, we proceed as in the estimate for the lower bound of the $\Vert \cdot \Vert _{H^{-k}({\mathbb {R}}^n)}$-norm, this time making use of (24):

26 $$\begin{aligned} \begin{aligned} \Vert \psi _N\Vert _{H^{t+s}({\mathbb {R}}^n)}&=\Vert \psi _N\Vert _{(H^{-(t+s)}({\mathbb {R}}^n))^*}\ge \frac{\Vert \psi _N\Vert ^2_{L^2({\mathbb {R}}^n)}}{\Vert \psi _N\Vert _{H^{-(t+s)}({\mathbb {R}}^n)}}\ge C_{t,s}N^{t+s-n/2}. \end{aligned} \end{aligned}$$

Combining (24), (25) and (26), we finally get the wanted estimate

27 $$\begin{aligned} C'_{t,s}N^{t+s-n/2}\le \Vert \psi _N\Vert _{H^{t+s}({\mathbb {R}}^n)}\le C_{t,s} N^{t+s-n/2}, \end{aligned}$$

holding for all $t\in {\mathbb {R}}$ such that t+s≥-M.

Using our sequence $(\psi _N)_{N\in {\mathbb {N}}}$ we will construct a new sequence $(\Phi _N)_{N\in {\mathbb {N}}}$ verifying the statement of the Lemma with $x_0=0$. Observe that we have $N^{s-n/2}\lesssim \Vert \psi _N\Vert _{H^s(\mathbb {R}^n)}\lesssim N^{s-n/2}$. Thus we can define

$$ \Phi _N:=\frac{\psi _N}{\Vert \psi _N\Vert _{H^s({\mathbb {R}}^n)}} $$

for all $N\in {\mathbb {N}}$. Trivially, we have $(\Phi _N)_{N\in {\mathbb {N}}}\in C^{\infty }_c({\mathbb {R}}^n)$, ${{\,\textrm{supp}\,}}(\Phi _N)\rightarrow \{0\}$ as $N\rightarrow \infty$ and

$$\begin{aligned} \Vert \Phi _N\Vert _{H^s({\mathbb {R}}^n)}=1. \end{aligned}$$

Morover, from (27) we get the estimates

$$\begin{aligned} \Vert \Phi _N\Vert _{H^{t+s}({\mathbb {R}}^n)}=\frac{\Vert \psi _N\Vert _{H^{t+s}({\mathbb {R}}^n)}}{\Vert \psi _N\Vert _{H^{s}({\mathbb {R}}^n)}} \le C_{t,s}N^{t+s-n/2}C_s^{-1}N^{-s+n/2}\le C_{t,s}N^t \end{aligned}$$

and

$$\begin{aligned} \Vert \Phi _N\Vert _{H^{t+s}({\mathbb {R}}^n)}=\frac{\Vert \psi _N\Vert _{H^{t+s}({\mathbb {R}}^n)}}{\Vert \psi _N\Vert _{H^{s}({\mathbb {R}}^n)}} \ge C_sN^{-s+n/2}C'_{t,s}N^{t+s-n/2}=C'_{t,s}N^{t} \end{aligned}$$

for all t+s≥-M.

Finally, assume $x_0\in \Omega _e$ is an arbitrary point, and let $N_0$ be the minimum element of ${\mathbb {N}}$ such that $x_0+[-1/N,1/N]^n\subset \Omega _e$. Define $\phi _N:= \Phi _{N+N_0}(\cdot -x_0)$ for all $N\in {\mathbb {N}}$. It is clear by construction that $\phi _N\in C^\infty _c(\Omega _e)$, with

$$\begin{aligned} \text{ supp }(\phi _N) \subset x_0 + \left[ -\frac{1}{N+N_0},\frac{1}{N+N_0}\right] ^n. \end{aligned}$$

Thus the new sequence $(\phi _N)_{N\in {\mathbb {N}}}$ verifies (iii). Moreover, as the $H^t({\mathbb {R}}^n)$ norms are translation invariant, we deduce that (i) and (ii) hold as well. □
